# Hepatic signal transducer and activator of transcription‐3 signalling drives early‐stage pancreatic cancer cachexia via suppressed ketogenesis

**DOI:** 10.1002/jcsm.13466

**Published:** 2024-04-17

**Authors:** Paige C. Arneson‐Wissink, Heike Mendez, Katherine Pelz, Jessica Dickie, Alexandra Q. Bartlett, Beth L. Worley, Stephanie M. Krasnow, Robert Eil, Aaron J. Grossberg

**Affiliations:** ^1^ Brenden‐Colson Center for Pancreatic Care Oregon Health & Science University Portland OR USA; ^2^ Division of Surgical Oncology, Department of Surgery, Knight Cancer Institute Oregon Health & Science University Portland OR USA; ^3^ Division of Oncological Sciences, Knight Cancer Institute Oregon Health & Science University Portland OR USA; ^4^ Department of Radiation Medicine Oregon Health & Science University Portland OR USA; ^5^ Cancer Early Detection Advanced Research Center Oregon Health & Science University Portland OR USA

**Keywords:** cachexia, interleukin‐6, ketogenesis, pancreatic cancer, STAT3

## Abstract

**Background:**

Patients with pancreatic ductal adenocarcinoma (PDAC) often suffer from cachexia, a wasting syndrome that significantly reduces both quality of life and survival. Although advanced cachexia is associated with inflammatory signalling and elevated muscle catabolism, the early events driving wasting are poorly defined. During periods of nutritional scarcity, the body relies on hepatic ketogenesis to generate ketone bodies, and lipid metabolism via ketogenesis is thought to protect muscle from catabolizing during nutritional scarcity.

**Methods:**

We developed an orthotopic mouse model of early PDAC cachexia in 12‐week‐old C57BL/6J mice. Murine pancreatic cancer cells (KPC) were orthotopically implanted into the pancreas of wild‐type, IL‐6^−/−^, and hepatocyte STAT3^−/−^ male and female mice. Mice were subject to fasting, 50% food restriction, ad libitum feeding or ketogenic diet interventions. We measured longitudinal body composition by EchoMRI, body mass and food intake. At the endpoint, we measured tissue mass, tissue gene expression by quantitative real‐time polymerase chain reaction, whole‐body calorimetry, circulating hormone levels, faecal protein and lipid content, hepatic lipid content and ketogenic response to medium‐chain fatty acid bolus. We assessed muscle atrophy in vivo and C2C12 myotube atrophy in vitro.

**Results:**

Pre‐cachectic PDAC mice did not preserve gastrocnemius muscle mass during 3‐day food restriction (−13.1 ± 7.7% relative to food‐restricted sham, *P* = 0.0117) and displayed impaired fatty acid oxidation during fasting, resulting in a hypoketotic state (ketogenic response to octanoate bolus, −83.0 ± 17.3%, *P* = 0.0328; *Hmgcs2* expression, −28.3 ± 7.6%, *P* = 0.0004). PDAC human patients display impaired fasting ketones (−46.9 ± 7.1%, *P* < 0.0001) and elevated circulating interleukin‐6 (IL‐6) (12.4 ± 16.5‐fold increase, *P* = 0.0001). IL‐6^−/−^ PDAC mice had improved muscle mass (+35.0 ± 3.9%, *P* = 0.0031) and ketogenic response (+129.4 ± 44.4%, *P* = 0.0033) relative to wild‐type PDAC mice. Hepatocyte‐specific signal transducer and activator of transcription 3 (STAT3) deletion prevented muscle loss (+9.3 ± 4.0%, *P* = 0.009) and improved fasting ketone levels (+52.0 ± 43.3%, *P* = 0.018) in PDAC mice. Without affecting tumour growth, a carbohydrate‐free diet improved tibialis anterior myofibre diameter (+16.5 ± 3.5%, *P* = 0.0089), circulating ketone bodies (+333.0 ± 117.6%, *P* < 0.0001) and *Hmgcs2* expression (+106.5 ± 36.1%, *P* < 0.0001) in PDAC mice. Ketone supplementation protected muscle against PDAC‐induced atrophy in vitro (+111.0 ± 17.6%, *P* < 0.0001 myofibre diameter).

**Conclusions:**

In early PDAC cachexia, muscle vulnerability to wasting is dependent on inflammation‐driven metabolic reprogramming in the liver. PDAC suppresses lipid β‐oxidation and impairs ketogenesis in the liver, which is reversed in genetically modified mouse models deficient in IL‐6/STAT3 signalling or through ketogenic diet supplementation. This work establishes a direct link between skeletal muscle homeostasis and hepatic metabolism. Dietary and anti‐inflammatory interventions that restore ketogenesis may be a viable preventative approach for pre‐cachectic patients with pancreatic cancer.

## Introduction

The wasting syndrome cachexia exacts a tremendous toll on both quality and duration of life among patients with cancer, and to date, there are no effective therapeutic strategies to prevent or reverse cachexia.^1^ Cancer cachexia is a complex behavioural and metabolic syndrome hallmarked by accelerated fat and muscle wasting, often in the context of nutritional deficit.^2^ Cachexia differs from pure undernutrition in two key ways: (1) Skeletal muscle is relatively spared during periods of undernutrition, but not in cachexia,^3^ and (2) nutritional supplementation is inadequate to reverse cachexia.^4^ Given these fundamental differences between undernutrition and cachexia, significant effort has been directed to understanding the paraneoplastic processes underlying the persistent and pervasive wasting associated with cachexia.

Pancreatic ductal adenocarcinoma (PDAC) is the cancer type most commonly associated with cachexia. Although nearly all patients with PDAC will develop cachexia at some point during their cancer journey, approximately half of these patients do not meet the diagnostic criteria for cachexia at the time of diagnosis.^5^ Cachexia is clinically defined by thresholds of weight and muscle loss^6^ and is closely tied to increased inflammatory cytokine signalling. Interleukin‐6 (IL‐6), in particular, is highly associated with PDAC progression, metastasis, mortality and cachexia in both patients and murine cancer models.^7^, ^8^, ^9^, ^10^, ^11^ ^,^ ^S1^ Anti‐IL‐6 therapies have had positive outcomes in rodents, but are not widely implemented clinically.^12^ ^,^ ^S2^ ^,^ ^S3^ This is potentially because most studies evaluating cancer cachexia compare patients or mice with fully developed cachexia against healthy control populations.^13^, ^14^, ^15^, ^16^, ^17^, ^18^ Thus, the early biology driving initial wasting, which may differ from the inflammation‐driven biology defined in more advanced cachexia, is not well characterized.^6^ Indeed, a recent study of early murine PDAC cachexia, using the autochthonous KPC model, showed no elevations in cachexia‐associated cytokines and was unable to discriminate cachexia from pure undernutrition.^19^ These data indicate that the processes canonically associated with cachexia may not be the driving processes most relevant during the pre‐cachectic state.

Maintaining metabolic homeostasis requires rapid adaptation to variable nutrient conditions. The liver is central to these adaptations by storing excess nutrition (fat and glycogen) during periods of nutritional abundance and shifting metabolism to gluconeogenesis and ketogenesis during times of low nutritional availability.^3^ ^,^ ^S4^ ^,^ ^S5^ Ketogenesis by the liver is hypothesized to be central to skeletal muscle preservation during fasting, because it relies on stored lipids, and not products of muscle proteolysis, to produce energy‐rich substrates that are used by other tissues.^3^ In humans and rodents undergoing prolonged starvation, exhaustion of adipose stores precipitates rapid muscle catabolism supporting the hypothesis that lipid mobilization protects muscle from catabolism when stored energy must be used.^20^ ^,^ ^S6^ Murine models of PDAC and lung cancer cachexia are associated with deficiencies in ketogenesis, implicating a role for the liver in the pathophysiology of cachexia.^11^, ^21^, ^22^ Though the liver is well positioned to coordinate altered metabolism in cachexia, its role remains poorly defined, particularly early in cancer cachexia, when wasting is initiated.

We propose that impaired ketogenesis is central to early PDAC cachexia development. We developed an orthotopic PDAC (OT‐PDAC) mouse model of early cachexia to define the events that mediate early‐stage tissue wasting and examine the roles of undernutrition, hepatic adaptive metabolism to undernutrition and inflammatory signalling in driving PDAC cachexia. Using this approach, we found that disrupted hepatic lipid metabolism is a key feature of early‐stage cachexia and that reversal of this deficit using two genetic models that block IL‐6 signalling, or a ketogenic diet (KD) intervention, was sufficient to prevent PDAC‐associated muscle loss.

## Methods

### Animal studies

#### Husbandry

C57BL/6J (wild‐type [WT], JAX 000664), B6.129S2‐*Il6* ^*tm1Kopf*^/J (IL‐6^−/−^, JAX 002650), B6N.Cg‐*Speer6‐ps1* ^*Tg(Alb‐cre)21Mgn*^/J (Alb‐Cre, JAX 018961), B6.129S1‐*Stat3* ^*tm1Xyfu*^/J (signal transducer and activator of transcription 3 [STAT3] fl/fl, JAX 016923) and STOCK *Gt(ROSA)26Sor* ^*tm4(ACTB‐tdTomato,‐EGFP)Luo*^/J (ROSA^mT/mG^, JAX 007576) mice were purchased from The Jackson Laboratory (Bar Harbor, ME) and maintained in our animal facility. *Kras* ^*G12D/+*^ *;p53* ^*R172H/+*^ *;Pdx*‐*Cre* (KPC) mice were provided as a gift from Rosalie Sears.^23^ All mice were housed and bred in a dedicated mouse room maintained at 26°C, 40% humidity and 12‐h light/dark cycle. Animals were provided with ad libitum access to food and water (5L0D, PicoLab) unless otherwise stated. All animals were 12 weeks of age at the start of the experiment. Sex is defined in the figure legends. All study animals were individually housed for acclimation at least 7 days prior to the procedure and for the duration of the study. All tumour studies followed humane endpoints. Mouse studies were conducted in accordance with the National Institutes of Health (NIH) Guide for the Care and Use of Laboratory Animals and approved by the Oregon Health & Science University (OHSU) Institutional Animal Care and Use Committee (IACUC). We performed OT‐PDAC implantations as previously described with modifications described in the Methods section of the supporting information.^15^ All animals were humanely euthanized via cardiac puncture under deep isoflurane anaesthesia.

#### Feeding schemes

##### Pair feeding

Beginning on Day 7, sham mice were pair‐fed to the mean weight of food consumed the prior day by PDAC animals.

##### Food restriction

The daily food allotment was calculated as 50% of their ad libitum consumption for 1 week prior to OT‐PDAC implantation. Mice were allowed to eat ad libitum for 3 days after surgery and then underwent food restriction (FR) for the final 4 days prior to euthanasia.

##### Fasting

Mice were moved into a clean cage, and food was withheld for the stated duration.

##### Ketogenic diet

We used the KD 93M diet (TD160153.PWD) and the nutritionally matched control 93M diet (CD, TD150345) from Envigo (Indianapolis, IN). Mice were pair‐fed based on caloric intake to the PDAC group within each diet.

### Human studies

Our study population included 37 patients diagnosed with PDAC who underwent surgical exploration or pancreatectomy at OHSU between 27 March 2012 and 28 June 2018. Detailed patient information is provided in *Table* *S1*. This study was approved by the OHSU institutional review board (IRB# 21923), and all patients provided informed consent. Patients were identified from an institutional database of 373 consecutive patients with potentially resectable PDAC. Skeletal muscle, visceral adipose tissue and subcutaneous adipose tissue areas were measured from a single axial slice at the L3 lumbar vertebral level of the pre‐surgical staging computed tomography scan.^24^ ^,^ ^S7^ Extended analysis details are provided in the supporting information.

### Cell lines

#### Growth conditions and validation

All cells were maintained at 37°C and 5% CO_2_ in a humidified incubator and tested negative in house for mycoplasma using the Universal Mycoplasma Detection Kit (30‐1012K). *Kras* ^*G12D/+*^ *;Tp53* ^*R172H/+*^ *;Pdx1‐Cre* (KPC) cell line was generously shared by Dr. Elizabeth Jaffee.^15^ ^,^ ^S8^ KPC cells were grown on tissue culture‐treated dishes in growth media consisting of RPMI 1640 (Gibco) with 10% foetal bovine serum (FBS) (Corning) and 1% penicillin/streptomycin (Gibco).

### Blood measurements

Prior to euthanasia, we used ketometers and glucometers to measure blood ketones (Keto‐Mojo GK+) and glucose (Ascensia Contour Next) in blood collected from tail‐nick.

### Echo magnetic resonance imaging body composition

Lean mass, fat mass, total body water and free water were measured using whole‐body magnetic resonance imaging (MRI) prior to tumour injection (Day 0) and then on Days 3, 7, 10 and 14 after tumour injection (EchoMRI, Houston, TX).

### Human plasma analytes

Fasting plasma β‐hydroxybutyrate (BHB) concentrations were measured using a colorimetric assay (Cayman Chemical #700190). Fasting plasma glucose was measured by a handheld glucometer (Ascensia Contour Next). Plasma IL‐6 was measured by immunoassay (ProQuantum Human IL‐6 Immunoassay Kit, Thermo Fisher) and run on the ABI 7300 (Applied Biosystems) with analyses performed using ProQuantum Software (Thermo Fisher).

### Immunostaining

Muscle tissue was prepared by placing it in a 30% sucrose sink overnight, cryofreezing in O.C.T. medium (Sakura) and sectioning at 8 μm on a cryostat at −18°C. We rehydrated sections in phosphate‐buffered saline (PBS), post‐fixed in 4% paraformaldehyde (PFA) for 15 min, permeabilized in 0.5% Triton X‐100 and blocked in 3% bovine serum albumin (BSA), 0.2% Triton X‐100 and 0.2% Tween 20 in PBS. Primary antibody incubations occurred at room temperature (RT) for 90 min, or 4°C overnight, followed by incubation with secondary antibodies at RT for 45 min. All fluorescent slides were counter‐stained with DAPI. Coverslips were mounted using ProLong Gold (Molecular Probes) or Fluoroshield (Abcam). We collected images of our stained tissues and cells using a Zeiss Axioskop and a Zeiss AxioCam MRm.

### Octanoate challenge

Mice in each experimental condition were fasted for 16 h overnight and injected intraperitoneally with sodium octanoate (Sigma), which was dissolved to 200 mM in 0.9% NaCl at a volume of 6 mL/kg.^11^ Blood ketones and glucose were monitored every 45 min at *t* = 0, 45, 90, 135 and 180 min after injection.

### Plasma analytes

Plasma concentrations of corticosterone (Thermo Fisher) and IL‐6 (BioLegend) were measured using an enzyme‐linked immunosorbent assay (ELISA) and read on a plate reader (BioTek). Plasma levels of IL‐6, insulin and glucagon were measured using a Milliplex multiplex magnetic bead immunoassay (Millipore) and read on the Luminex 200. Colorimetric assays were used to measure plasma levels of BHB (Cayman Chemical #700190) and acetoacetate (Abcam #ab180875).

### Quantitative real‐time polymerase chain reaction

We isolated RNA from cell pellets or tissue samples using the E.Z.N.A. Total RNA Kit I (Omega BioTek) and prepared cDNA using a high‐capacity cDNA reverse transcription kit (Applied Biosystems). Quantitative real‐time polymerase chain reaction (qPCR) was run on the ABI 7300 (Applied Biosystems) using TaqMan Fast Advanced PCR Master Mix (Applied Biosystems) or SYBR Green Master Mix (Applied Biosystems). The relative expression was calculated using the ΔΔC_t_ method.

### Western blotting

We extracted protein from snap‐frozen tissues by bead homogenization followed by brief sonication. Twenty micrograms of protein was loaded in each lane and run on 10–20% Tris–glycine gels (Invitrogen). Gels were transferred to polyvinylidene difluoride (PVDF) membranes (Millipore) and blocked with 5% BSA for 1 h. Membranes were incubated with primary antibodies overnight at 4°C with gentle agitation. Blots were then washed with Tris‐buffered saline with Tween 20 (TBST) and incubated in secondary antibodies for 1 h prior to imaging (LI‐COR Odyssey Imaging System).

### Statistical analysis

Specific statistical tests and sample size for each study are indicated in the figure legends. Error bars in figures show SEM. Statistical analyses were performed using GraphPad Prism (Version 9; GraphPad Software Inc.) or JMP Pro (Version 16; SAS Institute Inc.), and graphs were built using GraphPad Prism (GraphPad Software Inc.) statistical analysis software. *P* values are two‐sided with values <0.05 regarded as statistically significant.

### Data availability

Further information and resources, including plasmid sequences, engineered KPC cells and raw data, will be shared upon reasonable request to Aaron J. Grossberg (grossber@ohsu.edu).

## Results

### Pre‐cachexia is defined as muscle vulnerability to nutritional stress

**Figure 1:**
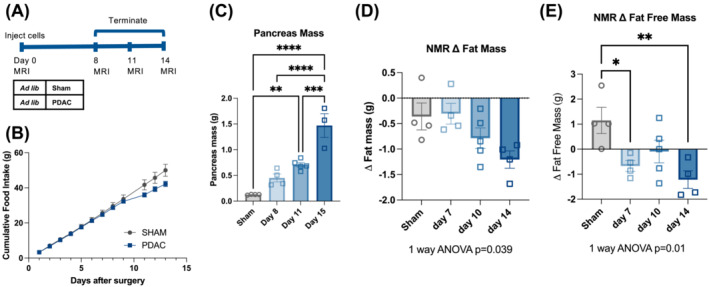
Orthotopic PDAC tumours induce cachexia. (A) Schematic for (B)–(E). *n* = 3 male (sham), 4 male (PDAC D8), 5 male (PDAC D11) and 4 male (PDAC D15). (B) Cumulative food intake for PDAC and sham animals. The mixed‐effects model showed statistical significance in the time/tumour interaction. *F*_interaction_(11, 74) = 5.822, *P* < 0.0001. (C) Pancreas mass across OT‐PDAC progression. Two PDAC mice euthanized prematurely were excluded from the tumour mass data. (D) EchoMRI measured fat mass relative to baseline across the OT‐PDAC progression. (E) EchoMRI measured fat‐free mass relative to baseline across the OT‐PDAC progression. Error bars represent SEM. Four‐group analyses were tested with a one‐way ANOVA and Tukey correction for multiple comparisons. *P < 0.05, ^**^ *P* < 0.01, ^***^ *P* < 0.001 and ^****^ *P* < 0.0001.

**Figure 2:**
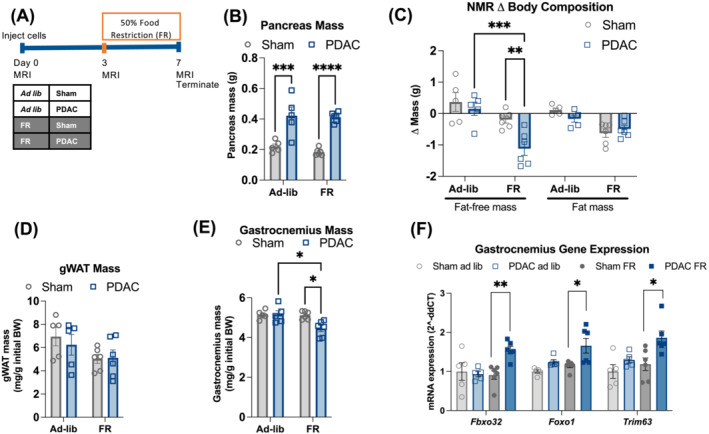
Pre‐cachexia is defined as skeletal muscle vulnerability to nutritional stress. (A) Schema of the food restriction (FR) pre‐cachexia model used in (B)–(F). *n* = 3 female and 2 male (sham ad libitum); 2 female and 4 male (sham FR); 3 female and 2 male (PDAC ad libitum); and 2 female and 4 male (PDAC FR). (B) Pancreas/tumour mass. (C) EchoMRI measured fat and fat‐free mass relative to baseline. (D) gWAT mass. (E) Gastrocnemius mass. (F) qPCR analysis of muscle atrophy genes measured in gastrocnemius muscle. Error bars represent SEM. All analyses for 2 × 2 studies were statistically tested with a full‐effects model, a two‐way ANOVA and the Šidák multiple comparisons test. *P < 0.05, ^**^ *P* < 0.01, ^***^ *P* < 0.001 and ^****^ *P* < 0.0001.

We modelled PDAC cachexia in mice using orthotopic implantation of PDAC cancer cells derived from the *Kras* ^*G12D/+*^ *;Tp53* ^*R172H/+*^ *;Pdx‐cre* (KPC) mouse into the pancreatic tail.^15^ Ad libitum‐fed PDAC‐bearing mice experience progressive wasting over the first 2 weeks of tumour development, which paralleled a decrease in food intake (*Figure* *1* *A*–*E*). We first questioned whether tissue wasting was attributable to mechanisms other than undernutrition and malabsorption, as previously suggested.^19^ If this was the case, we reasoned that equitable undernutrition in PDAC and sham animals should cause exacerbated wasting in the PDAC mice. To test this hypothesis, we restricted food intake to 50% of baseline (FR) in sham and PDAC mice for 4 days prior to euthanasia on Day 7 (*Figure* *2* *A*), a timepoint prior to when we see muscle wasting in ad libitum‐fed mice. FR did not affect the terminal size of pancreatic tumours (*Figure* *2* *B*). Although both sham and PDAC mice displayed decreased fat mass, FR elicited a loss of both fat‐free mass and gastrocnemius muscle mass in PDAC, but not sham, mice (*Figure* *2* *C*–*E*). Similarly, expression of atrophy‐associated genes *Fbxo32*, *Foxo1* and *Trim63* increased in gastrocnemius muscle from FR PDAC mice but not in ad libitum PDAC mice (*Figure* *2* *F*). Additionally, an acute 24‐h fast prior to euthanasia revealed a similar elevation in atrophy‐associated gene expression in PDAC compared to sham mice (*Figure* *S1* *A*). Together, these data show that PDAC mice exhibit increased vulnerability to two models of caloric deprivation, characterized by lean muscle wasting. Muscle wasting in PDAC mice was not associated with elevated basal metabolism, malabsorption, adipose browning or endocrine dysregulation (*Figure* *S1* *B*–*P*), all of which have been previously implicated in wasting pathologies.^19^, ^25^ ^,^ ^S9^

### Pancreatic ductal adenocarcinoma impairs the hepatic adaptive ketogenic response to fasting

**Figure 3:**
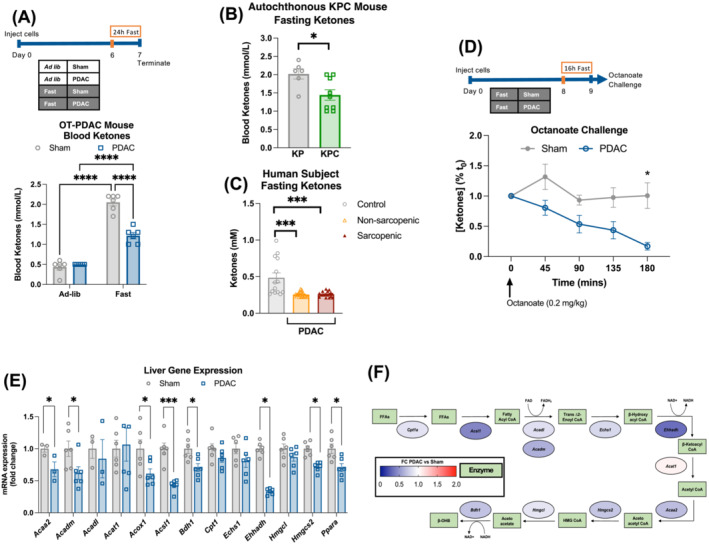
PDAC impairs the hepatic adaptive ketogenic response to fasting. (A) Blood ketone levels in ad libitum and fasted sham and PDAC mice with the schema of the pre‐cachexia model used in (A), (E) and (F). *n* = 3 female and 3 male mice per group. (B) Fasting ketones measured in autochthonous KPC mice and littermate KP controls, collected from 12‐ to 13‐week‐old mice after a 24‐h fast. *n* = 3 female and 3 male (KP); 7 female and 2 male (KPC). (C) Fasting ketones measured in plasma from non‐sarcopenic and sarcopenic patients with PDAC at the time of laparotomy. *n* = 14 control, 20 non‐sarcopenic PDAC and 18 sarcopenic PDAC with sarcopenia. Controls were collected from patients undergoing endoscopic ultrasound for non‐malignant indications. (D) Octanoate challenge blood ketone levels in sham and PDAC mice and schema for octanoate challenge after a 16‐h fast. *n* = 4 female and 4 male (PDAC); 3 female and 4 male (sham). (E) qPCR analysis of lipid metabolism genes measured in liver tissue. (F) Schematic depicting the relative expression of hepatic β‐oxidation and ketogenic genes from (E), with colour representing log_2_ fold‐change (PDAC vs. sham). Error bars represent SEM. All analyses for 2 × 2 studies were statistically tested with a full‐effects model, a two‐way ANOVA and the Šidák multiple comparisons test. A three‐group analysis was tested with a one‐way ANOVA and Tukey correction for multiple comparisons. A two‐group analysis was tested with an unpaired *t* test. *P < 0.05, ^**^ *P* < 0.01, ^***^ *P* < 0.001 and ^****^ *P* < 0.0001.

Lipid oxidation by the liver is postulated to be an essential mechanism for lean tissue sparing during fasting, as evidenced by the dependence of muscle preservation on adequate lipid stores and normal hepatic function.^3^ ^,^ ^S10^ As others have reported,^11^ we observed that PDAC impairs ketone release after fasting in both OT‐PDAC mice and the autochthonous PDAC, *Kras* ^*G12D/+*^ *;Tp53* ^*R172H/+*^ *;Pdx‐cre* (KPC) mouse model (*Figures* *3* *A*,*B* and *S2A*). We also found that patients with localized PDAC, who are instructed to fast for at least 8 h prior to resection, had lower blood ketones than healthy controls, regardless of whether they exhibited low skeletal muscle mass (*Figure* *3* *C* and *Table* *S1*). In OT‐PDAC mice, there was no difference in plasma acetoacetate: BHB, indicating no change in hepatocyte mitochondrial redox state^26^ (*Figure* *S2B*–*D*). In response to an octanoate challenge, we observed impaired ketogenic potential in PDAC mice, compared to sham^11^ ^,^ ^S11^ (*Figure* *3* *D*). At the transcriptional level, the expression of key enzymes involved in liver lipid import (*Cpt1*), β‐oxidation (*Acadm*, *Acadl*, *Acat1*, *Acox1*, *Acsl1*, *Echs1* and *Ehhadh*), ketogenesis (*Acaa2*, *Bdh1*, *Hmgcl* and *Hmgcs2*) and the master regulator of lipid metabolism, *Ppara*, were all decreased in the livers of PDAC mice, compared to sham (*Figure* *3* *E*,*F*). Although the expression of lipid oxidation genes is regulated by lipid availability, we observed no differences in plasma levels of triglycerides or non‐esterified fatty acids (NEFAs) (*Figure* *S2E*–*H*). Furthermore, we did not see differences in lipid content in gastrocnemius muscle or whole liver tissue, indicating that the decrease in β‐oxidation did not yield lipid buildup in either tissue (*Figure* *S2I*–*Q*). These data show that impairments in ketogenesis, a metabolic adaptation to undernutrition, are pervasive across both mouse models of PDAC and human patient populations.

### Impaired ketogenesis and cachexia in pancreatic ductal adenocarcinoma mice are interleukin‐6 dependent

**Figure 4:**
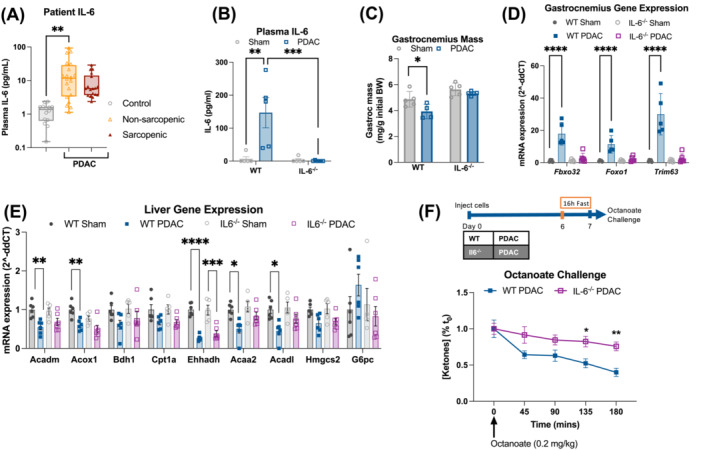
Impaired ketogenesis and cachexia in PDAC mice are IL‐6 dependent. (A) IL‐6 protein measured in plasma from control, non‐sarcopenic and sarcopenic patients with PDAC at the time of laparotomy. *n* = 14 control, 20 PDAC without sarcopenia and 18 PDAC with sarcopenia. Controls were collected from patients undergoing endoscopic ultrasound for non‐malignant indications. (B) IL‐6 concentration in plasma of WT and IL‐6^−/−^ mice. (C) Gastrocnemius mass at euthanasia, normalized to the initial body weight. (D) qPCR analysis of muscle atrophy genes measured in gastrocnemius muscle. (E) qPCR analysis of lipid metabolism genes measured in liver tissue at 10 days after tumour implantation in ad libitum‐fed mice. *n* = 3 female and 3 male (sham/WT); 3 female and 2 male (sham/IL‐6^−/−^); 3 female and 3 male (PDAC/WT); and 4 female and 2 male (PDAC/IL‐6^−/−^). (F) Blood ketone levels in WT and IL‐6^−/−^ PDAC mice in response to octanoate challenge after 16‐h fast and timeline schematic showing the 16‐h fast paradigm. *n* = 8 male mice per group. (B)–(E): *n* = 2 female and 3 male (sham/WT); 2 female and 3 male (sham/IL‐6^−/−^); 3 female and 2 male (PDAC/WT); and 3 female and 3 male (PDAC/IL‐6^−/−^). Error bars represent SEM. All analyses for 2 × 2 studies were statistically tested with a full‐effects model, two‐way ANOVA with Tukey correction for multiple comparisons. The octanoate challenge was statistically tested with repeated measures ANOVA with Šidák correction for multiple comparisons. Three‐group analyses were tested with a one‐way ANOVA and Tukey correction for multiple comparisons. Pairwise comparisons were tested with a parametric, unpaired *t* test. *P < 0.05, ^**^ *P* < 0.01, ^***^ *P* < 0.001 and ^****^ *P* < 0.0001. Independent biological replicates are indicated by individual points on bar graphs.

We were next interested in understanding the nature of the signal to the liver that drives metabolic reprogramming. Elevated circulating IL‐6 is associated with PDAC cachexia in patients and mice, and prior work showed restored ketogenesis in tumour‐bearing mice treated with IL‐6‐neutralizing antibody.^8^, ^9^, ^11^ We measured elevated IL‐6 in plasma from PDAC patients and our OT‐PDAC model (*Figure* *4* *A*,*B*). The KPC cells we used in OT‐PDAC did not produce or release IL‐6, and when we implanted these cells into IL‐6^−/−^ mice, we did not detect IL‐6 in the plasma (*Figures* *4* *B* and *S3A*). To identify the source of tumour‐associated IL‐6, we measured protein levels across tissues and found that pancreas/tumour tissue had the largest increase in IL‐6 protein between sham and OT‐PDAC mice (*Figure* *S3B*). Because very low levels of circulating IL‐6 are observed in sham mice, we interpreted the high but equivalent IL‐6 concentration in sham and PDAC liver and brown adipose tissue as reflecting intracellular cytokine stores. This led us to further investigate the intra‐tumoural cell population producing IL‐6. We identified podoplanin (PDPN)‐positive cancer‐associated fibroblasts, and CD45+ immune cells had the highest levels of IL‐6 and that CD45+ immune cells were the most abundant cell population quantified (*Figure* *S3C*,*D*). Within the CD45+ population, CD11b+ (myeloid) cells had the highest IL‐6 signal. We concluded that a mixed population of major histocompatibility complex II (MHCII)‐negative and MHCII‐positive myeloid cells are the major producers of IL‐6 in the PDAC microenvironment (*Figure* *S3E*,*F*).

To test whether whole‐body IL‐6 knockout (IL‐6^−/−^) mice would be refractory to cachexia development and ketogenic impairment, we implanted wild‐type (WT) and IL‐6^−/−^ mice with PDAC tumours and maintained them on an ad libitum diet for 11 days, at which timepoint WT mice have fully developed cachexia. There were no significant differences in food intake, body mass, tumour size or gonadal white adipose tissue (gWAT) mass across genotypes (*Figure* *S4A*–*D*). However, IL‐6^−/−^ mice were resistant to PDAC‐induced muscle wasting, as evidenced by the preservation of gastrocnemius muscle mass and decreased expression of muscle atrophy genes (*Fbxo32*, *Foxo1* and *Trim63*) (*Figure* *4* *C*,*D*). Together, these data demonstrate that IL‐6 is necessary for the development of muscle wasting in our PDAC model and broadly support prior research showing that IL‐6 is a major contributor to PDAC cachexia.^10^ We then evaluated hepatic metabolism in WT and IL‐6^−/−^ mice. Expression of hepatic genes involved in the initial steps of β‐oxidation (*Acadm*, *Acadl*, *Acox1* and *Acaa2*) had restored expression in IL‐6^−/−^ PDAC mice (*Figure* *4* *E*). Accordingly, both fasting ketone levels and ketogenic potential, as evaluated by the octanoate challenge, were increased in IL‐6^−/−^ PDAC mice, compared to WT (*Figures* *4* *F* and *S4E*). These data confirm that alterations in hepatic lipid metabolism in PDAC cachexia are dependent on IL‐6 signalling.

### Hepatic signal transducer and activator of transcription 3 mediates hepatic metabolic reprogramming and cachexia in pancreatic ductal adenocarcinoma mice

**Figure 5:**
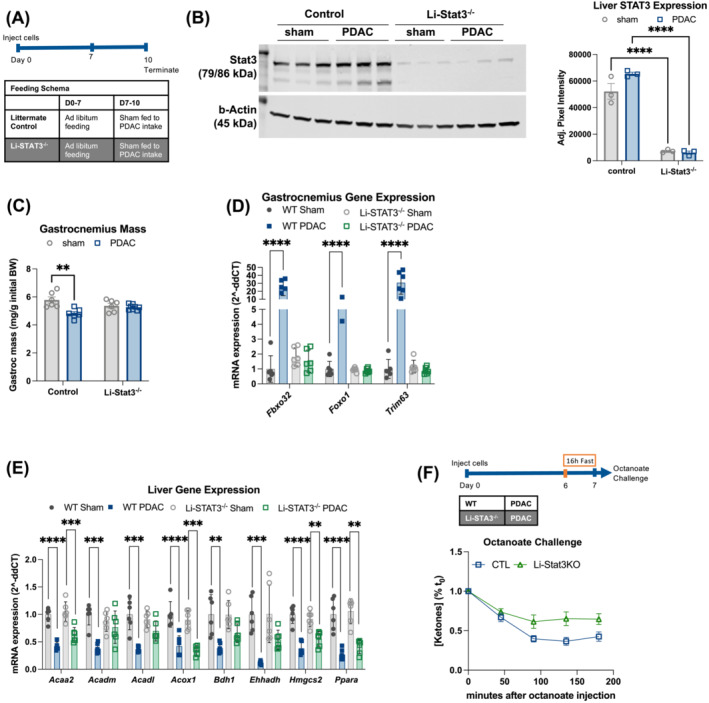
Hepatic STAT3 mediates hepatic metabolic reprogramming and cachexia in PDAC mice. (A) Schema for (B)–(E). *n* = 3 female and 3 male (sham/control); 2 female and 4 male (sham/Li‐*Stat3* ^−/−^); 2 female and 4 male (PDAC/control); and 3 female and 5 male (PDAC/Li‐*Stat3* ^−/−^). (B) Western blot for STAT3 (top) and β‐actin (bottom) and densitometry analysis of STAT3 western blot, normalized to β‐actin expression. (C) Gastrocnemius muscle, normalized to initial body weight. (D) qPCR analysis of muscle atrophy genes measured in gastrocnemius muscle. (E) qPCR analysis of lipid metabolism genes measured in liver tissue. (F) Blood ketone levels in PDAC mice in response to octanoate challenge and schema showing the 16‐h fast paradigm. *n* = 3 female and 3 male (PDAC/Li‐*Stat3* ^−/−^); 4 female and 3 male (PDAC/control). Time × genotype interaction, *F*(4, 44) = 4.09, *P* = 0.007. Error bars represent SEM. All analyses for 2 × 2 studies were statistically tested with a full‐effects model two‐way ANOVA with Tukey correction for multiple comparisons. The octanoate challenge was statistically tested with repeated measures ANOVA with Šidák correction for multiple comparisons. Pairwise comparisons were statistically tested with a parametric, unpaired *t* test. *P < 0.05, ^**^ *P* < 0.01, ^***^ *P* < 0.001 and ^****^ *P* < 0.0001. Independent biological replicates are indicated by individual points on bar graphs.

Because hepatocytes express the IL‐6 receptor (IL‐6R) and phosphorylate STAT3 in response to IL‐6,^27^ we hypothesized that IL‐6 exerts direct effects on hepatocytes to impair hepatic metabolism. We tested the effect of PDAC on liver metabolism and cachexia generation in hepatocyte‐specific *Stat3* knockout (*Alb‐Cre*/*Stat3* ^fl/fl^, referred to as Li‐*Stat3* ^−/−^) mice and pair‐fed littermate controls (*Figure* *5* *A*,*B*). Using ROSAmT/mG;*Alb‐Cre* reporter mice, we validated that Cre recombinase expression was restricted to hepatocytes (*Figure* *S5A*–*C*). Liver STAT3 phosphorylation and liver *Socs3* expression were increased in PDAC‐bearing littermate control mice but not in Li‐*Stat3* ^−/−^ mice, verifying that STAT3 activity was ablated in the livers of Li‐*Stat3* ^−/−^ mice even in the presence of an equivalent concentration of circulating IL‐6 (*Figure* *S5D*–*G*). We did not see any significant differences in cumulative food intake, body mass, tumour, gWAT or liver masses between genotypes (*Figure* *S5H*–*L*). Notably, hepatocyte‐specific STAT3 deletion completely reversed PDAC‐associated gastrocnemius muscle wasting and suppressed expression of muscle catabolism genes (*Fbxo32*, *Foxo1* and *Trim63*) (*Figure* *5* *C*,*D*). As in the IL‐6^−/−^ mice, we found that Li‐*Stat3* ^−/−^ PDAC mice had restored expression of several genes related to β‐oxidation and ketogenesis (*Acadm*, *Acadl*, *Bdh1* and *Ehhadh*) (*Figure* *5* *E*). Finally, we conducted an octanoate challenge after a 16‐h fast and found that Li‐*Stat3* ^−/−^ PDAC, compared to littermate control PDAC mice, had higher fasting blood ketones and improved ketogenic potential (*Figures* *5* *F* and *S5M*). These data demonstrate that hepatic reprogramming in response to IL‐6 is sufficient to drive PDAC cachexia.

### Ketogenic diet prevents muscle and liver dysfunction in pancreatic ductal adenocarcinoma cachexia

**Figure 6:**
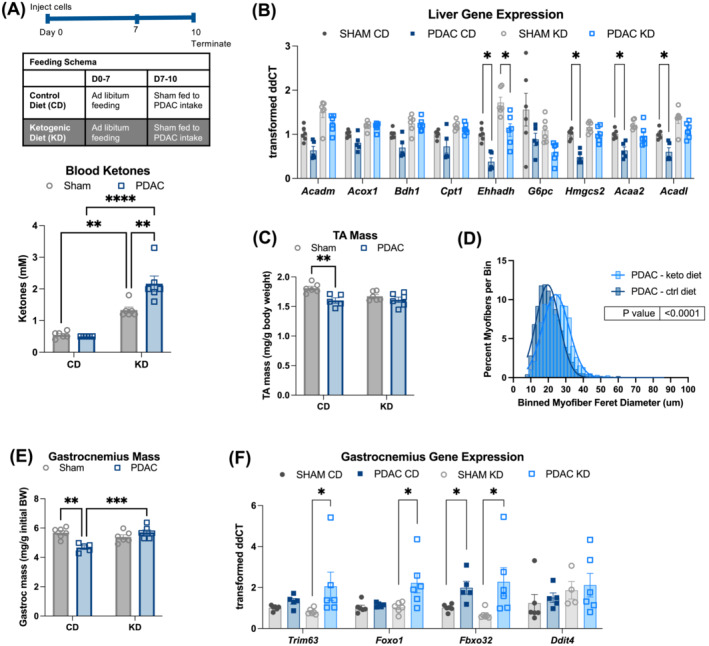
A ketogenic diet prevents hepatic metabolic reprogramming and cachexia in PDAC mice, independent of IL‐6. (A) Blood ketone levels at euthanasia and timeline schematic for the in vivo ketogenic diet study used in (A)–(F). *n* = 6 male mice (sham/CD, sham/KD and PDAC/KD) and 5 male mice (PDAC/CD). (B) qPCR analysis of lipid metabolism genes measured in liver tissue. (C) Tibialis anterior muscle mass normalized to initial body weight. (D) Quantification of the minimum Feret diameter of myofibres from PDAC mice fed with control or KD. Feret diameters were binned to a histogram and fit with a non‐linear regression (Gaussian, least squares regression). Myofibres in KD PDAC mice were significantly larger; *P* < 0.0001 by the extra sum‐of‐squares *F* test. (E) Gastrocnemius muscle mass normalized to the initial body weight. (F) qPCR analysis of muscle atrophy genes measured in gastrocnemius muscle. Error bars represent SEM. All analyses for 2 × 2 studies were statistically tested with a two‐way ANOVA with Tukey correction for multiple comparisons. *P < 0.05, ^**^ *P* < 0.01, ^***^ *P* < 0.001 and ^****^ *P* < 0.0001. Independent biological replicates are indicated by individual points on bar graphs.

Our observations that circulating ketones were lower across several PDAC datasets led us to question whether lower ketone levels were detrimental to muscle and if restored ketogenesis would be protective against cachexia. Although PDAC mice exhibit a deficit in fasting ketogenesis, we questioned whether chronic exposure to a KD would yield sufficient ketones to preserve muscle. We fed PDAC and sham mice with a carbohydrate‐free KD (*Tables* *S2* and *S3*), which was selected to maximally engage ketogenic metabolism or a calorically matched control diet for 10 days following implantation (*Figures* *6* *A* and *S6A*–*E* and *Table* *S3*). We pair‐fed sham mice to PDAC given the same diet to focus our study on tumour‐driven, rather than calorie‐driven effects on muscle and liver. KD did not affect the size of the tumour or plasma IL‐6 levels (*Figure* *S6G*,*H*). We also did not see differences in cumulative caloric intake, body mass, gross liver mass or gross gWAT mass between any of the groups (*Figure* *S6A*–*E*,*I*,*J*). As expected, KD induced elevated blood ketone levels, decreased blood glucose levels and increased plasma triglyceride levels (*Figure* *6* *A* and *S6K*,*L*). Next, we looked at the expression of hepatic lipid metabolism genes and found that KD restored the expression of several genes that were downregulated in control‐diet PDAC mice, including *Acadl*, a dehydrogenase essential for fatty acid oxidation; *Acaa2*, the final step in mitochondrial β‐oxidation; *Hmgcs2*, the rate‐limiting step in ketogenesis; and *Bdh1*, the interconversion step between the ketone bodies acetoacetate and BHB (*Figure* *6* *B*). The restoration of fasting ketone levels and hepatic gene expression indicate that the removal of carbohydrates from the diet is sufficient to reverse PDAC‐induced metabolic reprogramming. Furthermore, KD prevented PDAC‐associated muscle wasting, measured as both gastrocnemius and tibialis anterior (TA) gross muscle mass and TA myofibre cross‐sectional area (*Figures* *6* *C*–*E* and *S6M*–*O*). Paradoxically, KD did not reverse the increased expression of atrophy markers *Trim63*, *Foxo1* and *Fbxo32* in gastrocnemius muscle (*Figure* *6* *F*), which suggests that ketones exert pro‐anabolic effects even in the presence of increased catabolism in cachexia. We used cultured C2C12 myotubes to test whether ketones act directly on muscle to preserve muscle mass and found that BHB was sufficient to prevent myotube atrophy in myotubes treated with KPC cell conditioned medium (CM) (*Figure* *S7A*,*B*). While KPC CM treatment increases myotube expression of E3 ubiquitin ligases *Trim63* and *Fbxo32*, as we saw in mice fed with KD, increased expression of atrophy‐associated genes was not reversed with BHB treatment (*Figures* *6* *F* and *S7C*). These data indicate that ketone bodies are sufficient to prevent myotube atrophy without normalizing E3 ubiquitin ligase expression.

## Discussion

Although the aetiology of weight loss in PDAC is multifactorial, we identified an early cachexia phenotype, which is defined by the specific vulnerability of skeletal muscle to FR or fasting. FR was sufficient to induce muscle wasting in mice before overt symptoms of cachexia (muscle loss, weight loss or anorexia) were present. It is important to highlight that pure undernutrition (fasting or FR) did not cause muscle wasting in healthy mice. Sensitivity to FR in pre‐cachectic PDAC mice was not due to hypermetabolism or impaired nutrient absorption. Instead, the data pointed towards dysfunction in the adaptive hepatic metabolic response, implicating the liver as a central mediator of early cachexia.^25^ ^,^ ^S12^ Humans and mice are consistently cycling through fasting and feeding states over the course of the day, and extended periods of fasting are especially apparent during sleep hours.^28^ If the biology we describe herein is present in cachectic patients, this presents a daily fasting event to which they may be particularly vulnerable. The canonical metabolic response to fasting is to utilize stored lipids, and, as a result, humans experience the highest levels of blood ketones during their sleep hours.^29^ In the absence of lipid oxidation, the liver must utilize other substrates for gluconeogenic and ketogenic metabolism, such as glycogen and amino acids mobilized from the muscle.^3^ Many have speculated that a reliance on muscle breakdown to supply energy to the body is a contributor to cachexia progression, and our data provide one novel mechanism by which such a reliance could develop.^30^

Notably, *Stat3* deletion from hepatocytes was sufficient to reverse muscle catabolism and restore ketogenesis even in the presence of circulating IL‐6. Our results extend prior work by Flint et al. linking IL‐6 to impaired ketogenesis, providing the first direct evidence that hepatic ketogenesis is essential for muscle preservation in cachexia and defining the hepatocyte as the target for the anti‐ketogenic actions of IL‐6.^11^ Our two knockout approaches—whole‐body *Il‐6* ^−/−^ and Li‐*Stat3* ^*−/−*^—reveal the liver to be a novel target for IL‐6 signalling in cachexia. To our knowledge, this is also the first evidence that hepatic STAT3 activation could suppress lipid oxidation. Existing literature shows that constitutive hepatic STAT3 activation suppresses the gluconeogenic genes *Pck1* and *G6pc*, which are similarly upregulated in a low nutrient context.^31^ Despite the apparent deficit in β‐oxidation, PDAC mice did not exhibit fatty liver, which may be explained by the anti‐lipogenic effect of STAT3 activation, which can prevent steatosis in obesity models by inhibiting sterol regulatory element‐binding protein 1 (SREBP‐1).^32^ This provides evidence that STAT3 can transcriptionally repress lipid metabolism in the liver and that repressive control of hepatic adaptive metabolism is conserved across metabolic syndromes.

Ketogenesis is impaired in early‐stage cachexia, and the data presented here recapitulate previous reports indicating that increasing ketone production preserves skeletal muscle in murine models of cancer cachexia.^21^, ^22^ That ketones were sufficient to reverse myotube wasting in vitro suggests that ketones may exert anti‐catabolic effects directly on muscle, a concept supported by human data showing that infusion with BHB reduces muscle protein catabolism.^33^ Our results contradict recent reports showing that KD did not prevent cachexia in a KRAS‐driven murine lung cancer model, a heterotopic C26 colon cancer model or the autochthonous genetically engineered KPC PDAC model.^16^, ^34^, ^35^ Furthermore, KD delayed tumour growth in the latter two models, yet we observed no effect of KD on tumour size. These discrepant results could be explained by differences in the KD interventions or duration of KD feeding. First, in the prior studies, healthy mice were not calorically matched to cachectic mice, so wasting in tumour‐bearing mice may simply reflect a significant difference in calorie intake. Their observation that dexamethasone both increased KD intake and decreased cachexia reinforces the importance of sufficient nutritional intake and outlines the challenges of initiating KD intervention late in the course of cachexia development. The window to see a benefit in skeletal muscle preservation from KD may be limited, as our study only evaluated mice with early‐stage cachexia. Second, the prior studies used a low‐carbohydrate (1.8% kcal), low‐protein (4.7% kcal) KD, whereas we fed mice with a carbohydrate‐free diet with nearly double the caloric content provided by protein (9.5% kcal). The effects of the low‐carbohydrate, low‐protein diet on tumour growth and cachexia endpoints in the studies by Ferrer et al. could have been the result of protein malnutrition. Finally, the window to see a benefit in skeletal muscle preservation from KD may be limited, as our study only evaluated mice with early‐stage cachexia. Particularly in survival studies, if KD does not provide benefit to survival, it may be difficult to observe benefit in muscle retention at animals' humane endpoints. Similarly, if KD is effective in reducing tumour burden, we may not have observed this effect in our 10‐day studies when mice have not reached terminal‐stage tumour burden.

The heterogeneity in mouse models' response to KD highlights the complexity of introducing this as an anti‐cachectic therapeutic. Not all patients with cachexia exhibit elevated levels of IL‐6, and the levels of other cachexia mediators, such as growth and differentiation factor‐15 or tumour necrosis factor, are not uniformly elevated across all patients with cachexia.^7^ ^,^ ^S13^ ^,^ ^S14^ It is possible that different inflammatory states impact the efficacy of KD as a cachexia intervention. The beneficial effects of ketones may also be dose‐dependent or rely upon the specific nutritional constitution of the intervention. More work is needed to elucidate how feeding times, carbohydrate and protein content, and impaired digestion (pancreatic enzyme insufficiency) might impact the efficacy of KD.^36^ Although adherence to a KD is challenging for cancer patients, numerous clinical trials are investigating this intervention further.^37^ Despite these challenges, it is important to consider that retained muscle mass, even for a limited time early in cancer diagnosis, could provide substantial benefits to patients by improving quality of life and increasing the tolerability of anti‐tumour interventions.

There are several notable limitations of this study. First, we utilize an OT‐PDAC model with a relatively short experimental timeline. Although we are using this model to provide evidence for metabolic changes early in the development of cachexia, cachectic patients experience early‐stage cachexia for much longer than the 7–10 days represented in our studies. The limited timeline also limits our ability to provide evidence for the efficacy of KD for longer time frames in cachexia treatment. The FR paradigm we utilized was not chosen to replicate the conditions present in human patients but rather to place a challenge on the adaptive metabolic processes we hypothesized to be impaired in cachexia. Using this paradigm reflects strategies that are used in pre‐clinical and clinical settings to challenge various aspects of physiology (e.g., glucose tolerance test, adrenocorticotropic hormone [ACTH] simulation test and cardiac stress test), but does not provide a completely accurate representation of the patient experience. The effects of KD, IL‐6 knockout and hepatocyte‐specific *Stat3* knockout in cachexia development warrant further investigation in other models of PDAC cachexia. In addition to IL‐6, other activators of STAT3, such as leukaemia inhibitory factor (LIF), leptin and oncostatin M (OSM), may also meaningfully activate STAT3 in PDAC.^38^ We also did not consider how IL‐6R and IL‐6 trans‐signalling might contribute to cachexia development in our model, which would be an important future consideration as the liver is a source of IL‐6R and trans‐signalling is thought to mediate the pro‐inflammatory effects of IL‐6.^10^ Lastly, the relative effects of ketones on skeletal muscle catabolic and anabolic processes and the contribution of each to skeletal muscle preservation in our models remain unknown.

**Figure 7:**
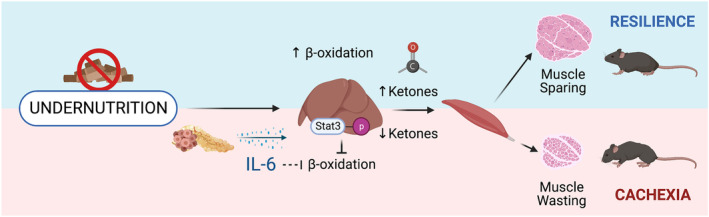
Summary of the main findings. Undernutrition leads to increased lipid oxidation in the liver, producing ketones as an energy source. This adaptive metabolic strategy spares muscle from breakdown during undernutrition. PDAC tumours secrete IL‐6, which signals through STAT3 in the liver to block ketogenesis. Loss of adaptive lipid metabolism leads to muscle loss in pre‐cachectic animals.

Early PDAC cachexia is defined by an increased susceptibility to FR orfasting, and a dysfunctional response to energy loss. Central to this is the failure of the liver to increase ketogenesis in PDAC mice (*Figure* *7*). We observed global downregulation of lipid oxidation genes in the livers of PDAC mice, which was reversed by three interventions: KD, whole‐body IL‐6 knockout and hepatocyte‐specific *Stat3* knockout. Our work defines a novel role for hepatic metabolic reprogramming in PDAC cachexia and identifies a new mechanism by which IL‐6 influences skeletal muscle wasting. Ultimately, this work highlights the importance of understanding nutritional and metabolic states in cachectic patients and provides evidence that strategies that improve ketogenesis could be valuable adjuvant interventions for cachectic patients.

## Conflict of interest statement

RE is a paid consultant and conducts ongoing research for Lyell Immunopharma. The other authors declare no competing interests.

## Funding

This work was supported by the National Cancer Institute (K08CA245188, R37CA280692 and R01CA264133), the Brenden‐Colson Center for Pancreatic Care, the Oregon Pancreas Tissue Registry, the Histopathology Shared Resource for Pathology Studies (University Shared Resource Program at Oregon Health and Science University [OHSU] Knight Cancer Institute [P30 CA069533 and P30 CA069533 13S5]) and the OHSU Flow Cytometry Shared Resource (OHSU Knight Cancer Institute NCI Cancer Center Support Grant P30 CA069533).

## Supporting information


**Figure S1.** OT‐PDAC cachexia progresses in the absence of aberrant energy balance, nutrient absorption, and adipose browning.
**Figure S2.** OT‐PDAC lowers lipid availability and does not cause lipid accumulation in liver.
**Table S1.** Characteristics of patients with pancreatic cancer.
**Figure S3.** IL‐6 is derived from the tumor microenvironment.
**Figure S4.** Physiology of IL‐6^‐/‐^ mice.
**Figure S5.** Hepatocyte STAT3 KO is tissue specific.
**Figure S6.** Nutrient intake and physiology of mice on ketogenic diet.
**Table S2.** Component by weight for ketogenic and control diets.
**Table S3.** Complete description of components included in ketogenic diet.
**Figure S7.** Ketones prevent myotube atrophy *in vitro*.
**Table S4.** Table of antibodies used.
**Table S5.** Table of qPCR TaqMan probes used.
**Table S6.** Table of qPCR Sybr primers used.

## References

[1] Fearon K , Arends J , Baracos V . Understanding the mechanisms and treatment options in cancer cachexia. Nat Rev Clin Oncol 2013;10:90–99. 23207794 10.1038/nrclinonc.2012.209

[2] Olson B , Zhu X , Norgard MA , Levasseur PR , Butler JT , Buenafe A , et al. Lipocalin 2 mediates appetite suppression during pancreatic cancer cachexia. Nat Commun 2021;12: 2057. 33824339 10.1038/s41467-021-22361-3 PMC8024334

[3] Cahill GF Jr . Starvation in man. N Engl J Med 1970;282:668–675. 4915800 10.1056/NEJM197003192821209

[4] Omlin A , Blum D , Wierecky J , Haile SR , Ottery FD , Strasser F . Nutrition impact symptoms in advanced cancer patients: frequency and specific interventions, a case–control study. J Cachexia Sarcopenia Muscle 2013;4:55–61. 23307589 10.1007/s13539-012-0099-x PMC3581613

[5] Kordes M , Larsson L , Engstrand L , Löhr JM . Pancreatic cancer cachexia: three dimensions of a complex syndrome. Br J Cancer 2021;124:1623–1636. 33742145 10.1038/s41416-021-01301-4 PMC8110983

[6] Fearon K , Strasser F , Anker SD , Bosaeus I , Bruera E , Fainsinger RL , et al. Definition and classification of cancer cachexia: an international consensus. Lancet Oncol 2011;12:489–495. 21296615 10.1016/S1470-2045(10)70218-7

[7] Ramsey ML , Talbert E , Ahn D , Bekaii‐Saab T , Badi N , Bloomston PM , et al. Circulating interleukin‐6 is associated with disease progression, but not cachexia in pancreatic cancer. Pancreatology 2019;19:80–87. 30497874 10.1016/j.pan.2018.11.002 PMC6613190

[8] Baltgalvis KA , Berger FG , Pena MM , Davis JM , Muga SJ , Carson JA . Interleukin‐6 and cachexia in *Apc* ^Min/+^ mice. Am J Physiol Regul Integr Comp Physiol 2008;294:R393–R401. 18056981 10.1152/ajpregu.00716.2007

[9] Bonetto A , Aydogdu T , Jin X , Zhang Z , Zhan R , Puzis L , et al. JAK/STAT3 pathway inhibition blocks skeletal muscle wasting downstream of IL‐6 and in experimental cancer cachexia. Am J Physiol‐Endocrinol Metab 2012;303:E410–E421. 22669242 10.1152/ajpendo.00039.2012 PMC3423125

[10] Rupert JE , Narasimhan A , Jengelley DH , Jiang Y , Liu J , Au E , et al. Tumor‐derived IL‐6 and trans‐signaling among tumor, fat, and muscle mediate pancreatic cancer cachexia. J Exp Med 2021;218:e20190450. 33851955 10.1084/jem.20190450 PMC8185651

[11] Flint TR , Janowitz T , Connell CM , Roberts EW , Denton AE , Coll AP , et al. Tumor‐induced IL‐6 reprograms host metabolism to suppress anti‐tumor immunity. Cell Metab 2016;24:672–684. 27829137 10.1016/j.cmet.2016.10.010 PMC5106372

[12] Chen I , Johansen J , Zimmers T , Dehlendorff C , Parner VK , Jensen BV , et al. PACTO: a single center, randomized, phase II study of the combination of nab‐paclitaxel and gemcitabine with or without tocilizumab, an IL‐6R inhibitor, as first‐line treatment in patients with locally advanced or metastatic pancreatic cancer. Ann Oncol 2017;28:v266.

[13] Talbert EE , Cuitino MC , Ladner KJ , Rajasekerea PV , Siebert M , Shakya R , et al. Modeling human cancer‐induced cachexia. Cell Rep 2019;28:1612–1622.e4. 31390573 10.1016/j.celrep.2019.07.016 PMC6733019

[14] Arneson‐Wissink PC , Ducharme AM , Doles JD . A novel transplantable model of lung cancer‐associated tissue loss and disrupted muscle regeneration. Skeletal Muscle 2020;10:6. 32151276 10.1186/s13395-020-00225-6 PMC7063717

[15] Michaelis KA , Zhu X , Burfeind KG , Krasnow SM , Levasseur PR , Morgan TK , et al. Establishment and characterization of a novel murine model of pancreatic cancer cachexia. J Cachexia Sarcopenia Muscle 2017;8:824–838. 28730707 10.1002/jcsm.12225 PMC5659050

[16] Langer HT , Ramsamooj S , Liang RJ , Grover R , Hwang S‐K , Goncalves MD . Systemic ketone replacement does not improve survival or cancer cachexia in mice with lung cancer. Front Oncol 2022;12:12. 10.3389/fonc.2022.903157 PMC9203842 35719965

[17] Braun TP , Grossberg AJ , Krasnow SM , Levasseur PR , Szumowski M , Zhu XX , et al. Cancer‐ and endotoxin‐induced cachexia require intact glucocorticoid signaling in skeletal muscle. FASEB J 2013;27:3572–3582. 23733748 10.1096/fj.13-230375 PMC3752537

[18] Zhu X , Burfeind KG , Michaelis KA , Braun TP , Olson B , Pelz KR , et al. MyD88 signalling is critical in the development of pancreatic cancer cachexia. J Cachexia Sarcopenia Muscle 2019;10:378–390. 30666818 10.1002/jcsm.12377 PMC6463469

[19] Danai LV , Babic A , Rosenthal MH , Dennstedt EA , Muir A , Lien EC , et al. Altered exocrine function can drive adipose wasting in early pancreatic cancer. Nature 2018;558:600–604. 29925948 10.1038/s41586-018-0235-7 PMC6112987

[20] Flatt JP , Quail JM . Effects of liver damage on ketone‐body production and nitrogen balance in starved rats. Biochem J 1981;198:227–230. 7325997 10.1042/bj1980227 PMC1163232

[21] Shukla SK , Gebregiworgis T , Purohit V , Chaika NV , Gunda V , Radhakrishnan P , et al. Metabolic reprogramming induced by ketone bodies diminishes pancreatic cancer cachexia. Cancer Metab 2014;2:1–19. 25228990 10.1186/2049-3002-2-18 PMC4165433

[22] Goncalves MD , Hwang S‐K , Pauli C , Murphy CJ , Cheng Z , Hopkins BD , et al. Fenofibrate prevents skeletal muscle loss in mice with lung cancer. Proc Natl Acad Sci 2018;115:E743–E752. 29311302 10.1073/pnas.1714703115 PMC5789923

[23] Hingorani SR , Wang L , Multani AS , Combs C , Deramaudt TB , Hruban RH , et al. Trp53R172H and KrasG12D cooperate to promote chromosomal instability and widely metastatic pancreatic ductal adenocarcinoma in mice. Cancer Cell 2005;7:469–483. 15894267 10.1016/j.ccr.2005.04.023

[24] Grossberg AJ , Chamchod S , Fuller CD , Mohamed AS , Heukelom J , Eichelberger H , et al. Association of body composition with survival and locoregional control of radiotherapy‐treated head and neck squamous cell carcinoma. JAMA Oncol 2016;2:782–789. 26891703 10.1001/jamaoncol.2015.6339 PMC5080910

[25] Lieffers JR , Mourtzakis M , Hall KD , McCargar LJ , Prado CM , Baracos VE . A viscerally driven cachexia syndrome in patients with advanced colorectal cancer: contributions of organ and tumor mass to whole‐body energy demands. Am J Clin Nutr 2009;89:1173–1179. 19244378 10.3945/ajcn.2008.27273 PMC2667460

[26] Satapati S , Kucejova B , Duarte JA , Fletcher JA , Reynolds L , Sunny NE , et al. Mitochondrial metabolism mediates oxidative stress and inflammation in fatty liver. J Clin Invest 2015;125:4447–4462. 26571396 10.1172/JCI82204 PMC4665800

[27] Lee JW , Stone ML , Porrett PM , Thomas SK , Komar CA , Li JH , et al. Hepatocytes direct the formation of a pro‐metastatic niche in the liver. Nature 2019;567:249–252. 30842658 10.1038/s41586-019-1004-y PMC6430113

[28] Gill S , Panda S . A smartphone app reveals erratic diurnal eating patterns in humans that can be modulated for health benefits. Cell Metab 2015;22:789–798. 26411343 10.1016/j.cmet.2015.09.005 PMC4635036

[29] Masi D , Spoltore ME , Rossetti R , Watanabe M , Tozzi R , Caputi A , et al. The influence of ketone bodies on circadian processes regarding appetite, sleep and hormone release: a systematic review of the literature. Nutrients 2022;14:1410. 35406023 10.3390/nu14071410 PMC9002750

[30] Leij‐Halfwerk S , Dagnelie PC , van den Berg JWO , Wattimena JDL , Hordijk‐Luijk CH , Wilson JP . Weight loss and elevated gluconeogenesis from alanine in lung cancer patients. Am J Clin Nutr 2000;71:583–589. 10648275 10.1093/ajcn/71.2.583

[31] Inoue H , Ogawa W , Ozaki M , Haga S , Matsumoto M , Furukawa K , et al. Role of STAT‐3 in regulation of hepatic gluconeogenic genes and carbohydrate metabolism in vivo. Nat Med 2004;10:168–174. 14716305 10.1038/nm980

[32] Gurzov EN , Stanley WJ , Pappas EG , Thomas HE , Gough DJ . The JAK/STAT pathway in obesity and diabetes. FEBS J 2016;283:3002–3015. 26972840 10.1111/febs.13709

[33] Thomsen HH , Rittig N , Johannsen M , Møller AB , Jørgensen JO , Jessen N , et al. Effects of 3‐hydroxybutyrate and free fatty acids on muscle protein kinetics and signaling during LPS‐induced inflammation in humans: anticatabolic impact of ketone bodies. Am J Clin Nutr 2018;108:857–867. 30239561 10.1093/ajcn/nqy170

[34] Ferrer M , Mourikis N , Davidson EE , Kleeman SO , Zaccaria M , Habel J , et al. Ketogenic diet promotes tumor ferroptosis but induces relative corticosterone deficiency that accelerates cachexia. Cell Metab 2023;35:1147–62.e7. 37311455 10.1016/j.cmet.2023.05.008 PMC11037504

[35] Koutnik AP , Poff AM , Ward NP , DeBlasi JM , Soliven MA , Romero MA , et al. Ketone bodies attenuate wasting in models of atrophy. J Cachexia Sarcopenia Muscle 2020;11:973–996. 32239651 10.1002/jcsm.12554 PMC7432582

[36] Cortez NE , Mackenzie GG . Ketogenic diets in pancreatic cancer and associated cachexia: cellular mechanisms and clinical perspectives. Nutrients 2021;13:3202. 34579079 10.3390/nu13093202 PMC8471358

[37] Chung H‐Y , Park YK . Rationale, feasibility and acceptability of ketogenic diet for cancer treatment. J Cancer Prev 2017;22:127–134. 29018777 10.15430/JCP.2017.22.3.127 PMC5624453

[38] Zimmers TA , Fishel ML , Bonetto A . STAT3 in the systemic inflammation of cancer cachexia. Semin Cell Dev Biol 2016;54:28–41. 26860754 10.1016/j.semcdb.2016.02.009 PMC4867234

[39] von Haehling S , Morley JE , Coats AJS , Anker SD . Ethical guidelines for publishing in the Journal of Cachexia, Sarcopenia and Muscle: update 2021. J Cachexia Sarcopenia Muscle 2021;12:2259–2261. 34904399 10.1002/jcsm.12899 PMC8718061

