# Correction to ‘Hepatic signal transducer and activator of transcription‐3 signalling drives early‐stage pancreatic cancer cachexia via suppressed ketogenesis’

**DOI:** 10.1002/jcsm.13687

**Published:** 2024-12-26

Arneson‐Wissink P. C., Mendez H., Pelz K., Dickie J., Bartlett A. Q., Worley B. L., et al (2024) Hepatic signal transducer and activator of transcription‐3 signalling drives early‐stage pancreatic cancer cachexia via suppressed ketogenesis, *Journal of Cachexia, Sarcopenia and Muscle*. https://doi.org/10.1002/jcsm.13466.

In our attribution of funding, we mistakenly omitted the following: PCAW was supported by the National Cancer Institute K99CA286709–01.

We apologize for this error.

