# Supplementary material for: Hepatic signal transducer and activator of transcription‐3 signalling drives early‐stage pancreatic cancer cachexia via suppressed ketogenesis
Source: J Cachexia Sarcopenia Muscle. 2024 Apr 17;15(3):975–88. doi: 10.1002/jcsm.13466 (PMC11154744; doi:10.1002/jcsm.13466)
Supplement: Supplementary file 1 — Figure S1. OT‐PDAC cachexia progresses in the absence of aberrant energy balance, nutrient absorption, and adipose browning. Figure S2. OT‐PDAC lowers lipid availability and does not cause lipid accumulation in liver. Table S1. Characteristics of patients with pancreatic cancer. Figure S3. IL‐6 is derived from the tumor microenvironment. Figure S4. Physiology of IL‐6‐/‐ mice. Figure S5. Hepatocyte STAT3 KO is tissue specific. Figure S6. Nutrient intake and physiology of mice on ketogenic diet. Table S2. Component by weight for ketogenic and control diets. Table S3. Complete description of components included in ketogenic diet. Figure S7. Ketones prevent myotube atrophy in vitro. Table S4. Table of antibodies used. Table S5. Table of qPCR TaqMan probes used. Table S6. Table of qPCR Sybr primers used. [file JCSM-15-975-s001.pdf]

Supplemental Figure 1

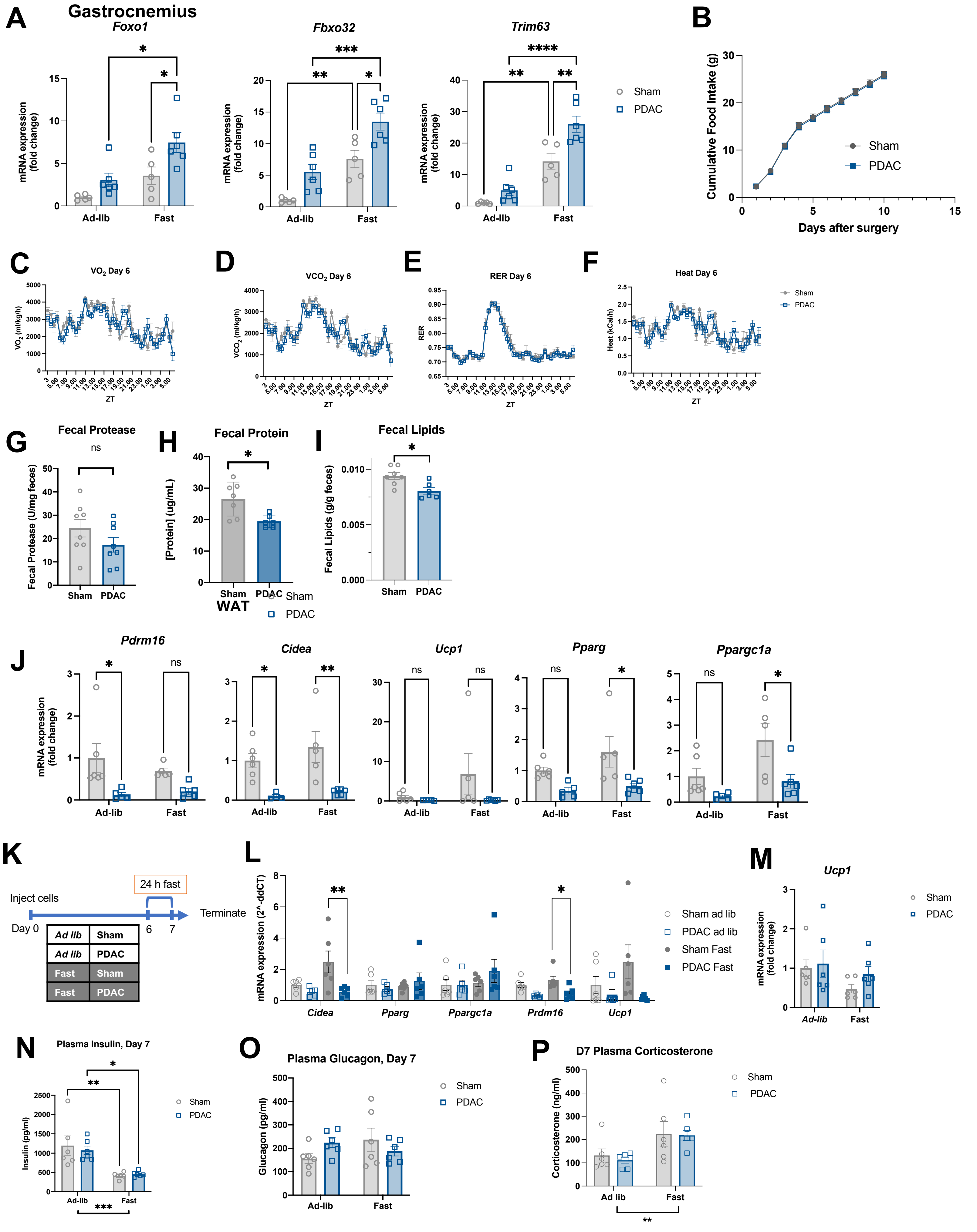

Supplemental Figure 1. OT-PDAC cachexia progresses in the absence of aberrant energy balance, nutrient absorption, and adipose browning. (A) qPCR analysis of muscle atrophy genes (*Foxo1*, *Fbxo32*, *Trim63*) measured in gastrocnemius muscle after 16 h fast, 11 d post implantation. n = 5 males (sham groups), 6 males (PDAC groups). (B) Cumulative food intake for indirect calorimetry experiment. Mice were fed ad lib D0-D6, and pair fed to PDAC mice D7-10. (C-F) Indirect calorimetry during d 6 post-implantation, representing final 24 hours before termination timepoint. n = 4 male and 4 female mice per group. (C) VO2, (D) VCO2, (E) respiratory exchange ratio (RER), (F) heat output. (G) Fecal protease activity, (H) fecal protein content, and (I) fecal lipid content, normalized to mass of feces of sham and PDAC animals fed ad libitum for 7 days. n = 4 female, 4 male mice per group (G); n = 3 female, 4 male (sham), 3 female, 3 male (PDAC) (H-I). (J) qPCR analysis of adipose browning genes (*Cidea*, *Pparg*, *Ppargc1a*, *Prdm16*, *Ucp1*) measured in white adipose tissue, 10 days post implantation after 24 hour fast. n = 6 male mice per group. (K) Schema of the 24 h fast pre-cachexia model used in panels M-T. n = 3 female, 3 male mice per group (L-P). (L) qPCR analysis of adipose browning genes (*Cidea*, *Pparg*, *Ppargc1a*, *Prdm16*, *Ucp1*) measured in white adipose tissue. (M) *Ucp1* expression in brown adipose tissue (BAT). (N) Terminal plasma insulin, (O) glucagon, and (P) corticosterone measured in *ad libitum* and fasted sham and PDAC mice. Error bars represent SEM. All analyses for 2x2 studies were statistically tested with a full effects model 2-way ANOVA and Sidak multiple comparisons test. 4-group analyses tested with one-way ANOVA and Tukey correction for multiple comparisons. **** $p<0.0001$, ***$p<0.001$, **$p<0.01$, *$p<0.05$.

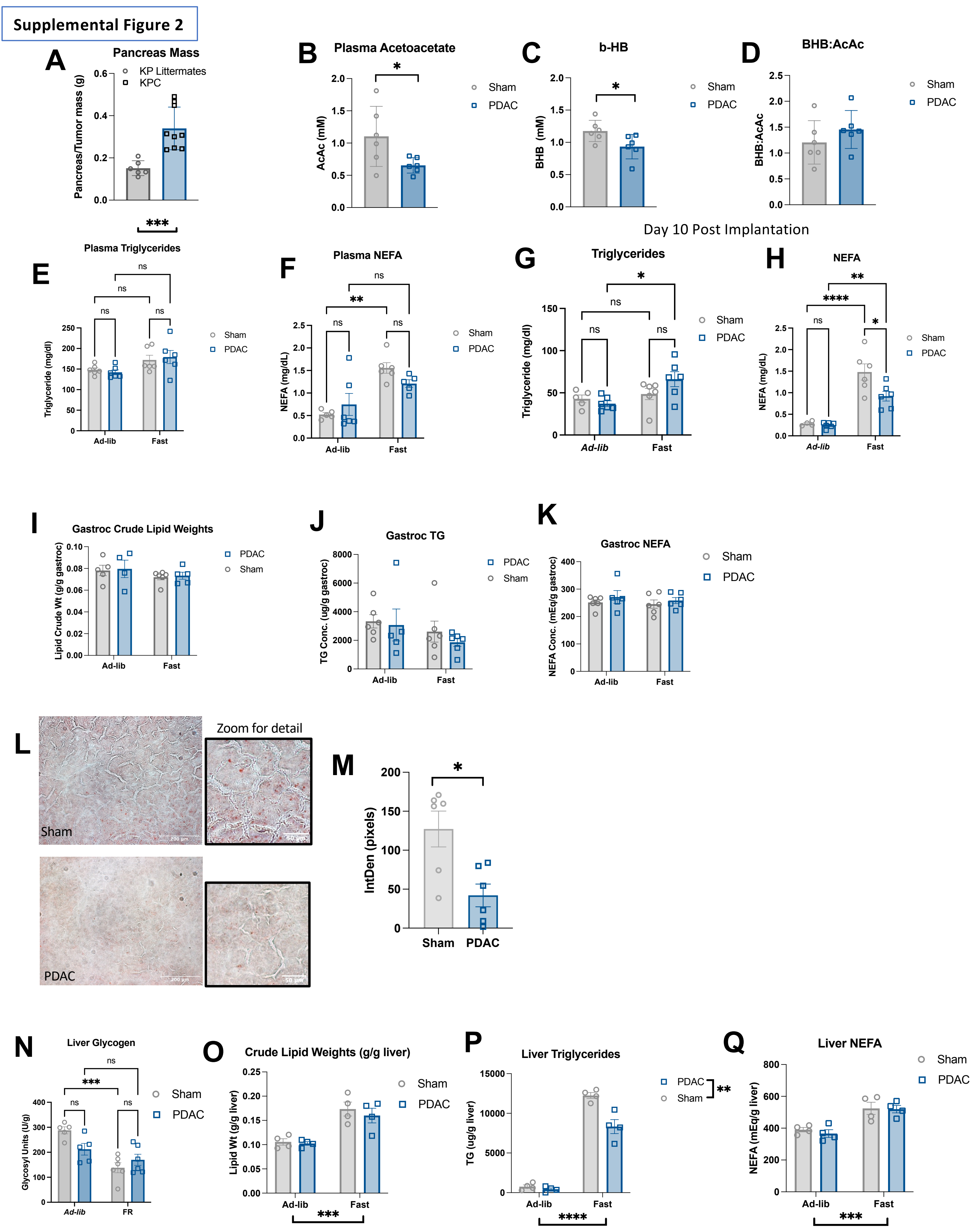
Supplemental Figure 2
A
Pancreas Mass
KP Littermates
KPC
Pancreas/Tumor mass (g)
***
B
Plasma Acetoacetate
AcAc (mM)
Sham
PDAC
*
C
b-HB
BHB (mM)
D
BHB:AcAc
Day 10 Post Implantation
E
Plasma Triglycerides
Triglyceride (mg/dl)
Ad-lib
Fast
ns
F
Plasma NEFA
NEFA (mg/dL)
**
G
Triglycerides
Triglyceride (mg/dl)
H
NEFA
NEFA (mg/dL)
****
I
Gastroc Crude Lipid Weights
Lipid Crude Wt (g/g gastroc)
J
Gastroc TG
TG Conc. (ug/g gastroc)
K
Gastroc NEFA
NEFA Conc. (mEq/g gastroc)
L
Zoom for detail
Sham
PDAC
M
IntDen (pixels)
N
Liver Glycogen
Glycosyl Units (U/g)
FR
O
Crude Lipid Weights (g/g liver)
Lipid Wt (g/g liver)
P
Liver Triglycerides
TG (ug/g liver)
Q
Liver NEFA
NEFA (mEq/g liver)

Supplemental Figure 2. OT-PDAC lowers lipid availability and does not cause lipid accumulation in liver. (A) Pancreas mass in KPC autochthonous and KP littermate control mice at 12-13 weeks. (B) Plasma acetoacetate levels (mM) in sham and PDAC mice. (C) Plasma BHB levels (mM) in sham and PDAC mice. (D) Ratio of plasma BHB and acetoacetate levels in sham and PDAC mice. (B-D) n = 6 male mice per group. (E) Plasma triglycerides (mg/dL) and (F) plasma NEFA (mg/dL) at 7 days post OT-PDAC implantation. (E-F) 3 female, 3 male mice per group. (G) Plasma triglycerides (mg/dL) and (H) plasma NEFA (mg/dL) at 10 days post OT-PDAC implantation. (G-H) n = 6 male mice per group. (I) Crude lipid weight, (J) triglyceride content, and (K) NEFA content in gastrocnemius muscle tissue, normalized to total tissue mass. (I-K) n = 3 female, 3 male (sham groups); 2 female, 3 male (PDAC groups). (L) Oil Red-O staining of liver from sham (top) and PDAC (bottom) with zoom inset to show lipid droplets. (M) Quantification of Oil Red-O staining in liver, measured as integrated density by pixels. n = 3 males, 3 females per group. (N) Liver glycogen content per g of tissue. n = 3 female, 2 male (sham/ad lib); 2 female, 4 male (sham/FR); 3 female, 2 male (PDAC/ad lib); 2 female, 4 male (PDAC/FR). (O) Crude lipid weight, (P) triglyceride content, and (Q) NEFA content in liver tissue, normalized to total tissue mass. (N-P) n = 4 male mice per group. Error bars represent SEM. All analyses for 2x2 studies were statistically tested with a full effects model 2-way ANOVA and Sidak multiple comparisons test. Asterisks below the x axis or in the legend indicate significant group effects. Pairwise comparisons statistically tested with parametric, unpaired t-test. **** $p<0.0001$, ***$p<0.001$, **$p<0.01$, *$p<0.05$.

Characteristic	Control	Sarcopenia No	Sarcopenia Yes	*P*-value
No. of patients	14	20	18	
Mean age at diagosis, years	62.4 (13.2)	62.5 (11.4)	73.6 (9.1)	*0.005*
Sex (%)				0.67
Female	7 (50)	13 (65)	10 (56)	
Male	7 (50)	7 (35)	8 (44)	
Race (%)				*0.02*
Non-white	4 (29)	1 (5)	0 (0)	
White	10 (71)	19 (95)	18 (100)	
Diagnosis				*<0.0001*
Adenocarcinoma		20 (100)	20 (100)	
Acute pancreatitis	3 (21)			
Chronic pancreatitis	5 (36)			
Benign gangioneuroma	1 (7)			
Non-malignant mass	1 (7)			
Polymorphous lymphocytic population	1 (7)			
Serous cystadenoma	3 (21)			
Stage (%)				0.11
Localized		3 (15)	8 (44)	
Regional		14 (70)	9 (50)	
Metastatic		3 (15)	1 (6)	
Grade (%)				0.80
Well-differentiated		1 (5)	1 (6)	
Moderately differentiated		10 (50)	8 (44)	
Poorly differentiated		4 (20)	6 (33)	
Undetermined		5 (25)	3 (17)	
Mean CA19-9 at time of surgery (SD)		1036 (2740)	1154 (3327)	0.22
Median OS (mo)	Not reached	18.1	17.4	0.35
BMI (kg/m^2)	28.2 (5.0)	30.1 (4.5)	25.3 (4.8)	*0.01*
L3 SMI (cm^2/m^2)		50.8 (1.7)	37.9 (1.7)	*<0.0001*
L3 Muscle CSA (cm^2)		142.6 (6.6)	108.8 (6.8)	*0.001*
L3 Subcutaneous fat CSA (cm^2)		259.5 (24.4)	172.9 (27.5)	*0.02*
L3 Visceral fat CSA (cm^2)		160.6 (19.1)	121.4 (19.6)	0.16

Supplemental Table 1. Characteristics of patients with pancreatic cancer. Continuous variables are reported as mean (SD). Categorical variables reported as number (percentage) at the time of surgery, unless otherwise noted.

Supplemental Figure 3

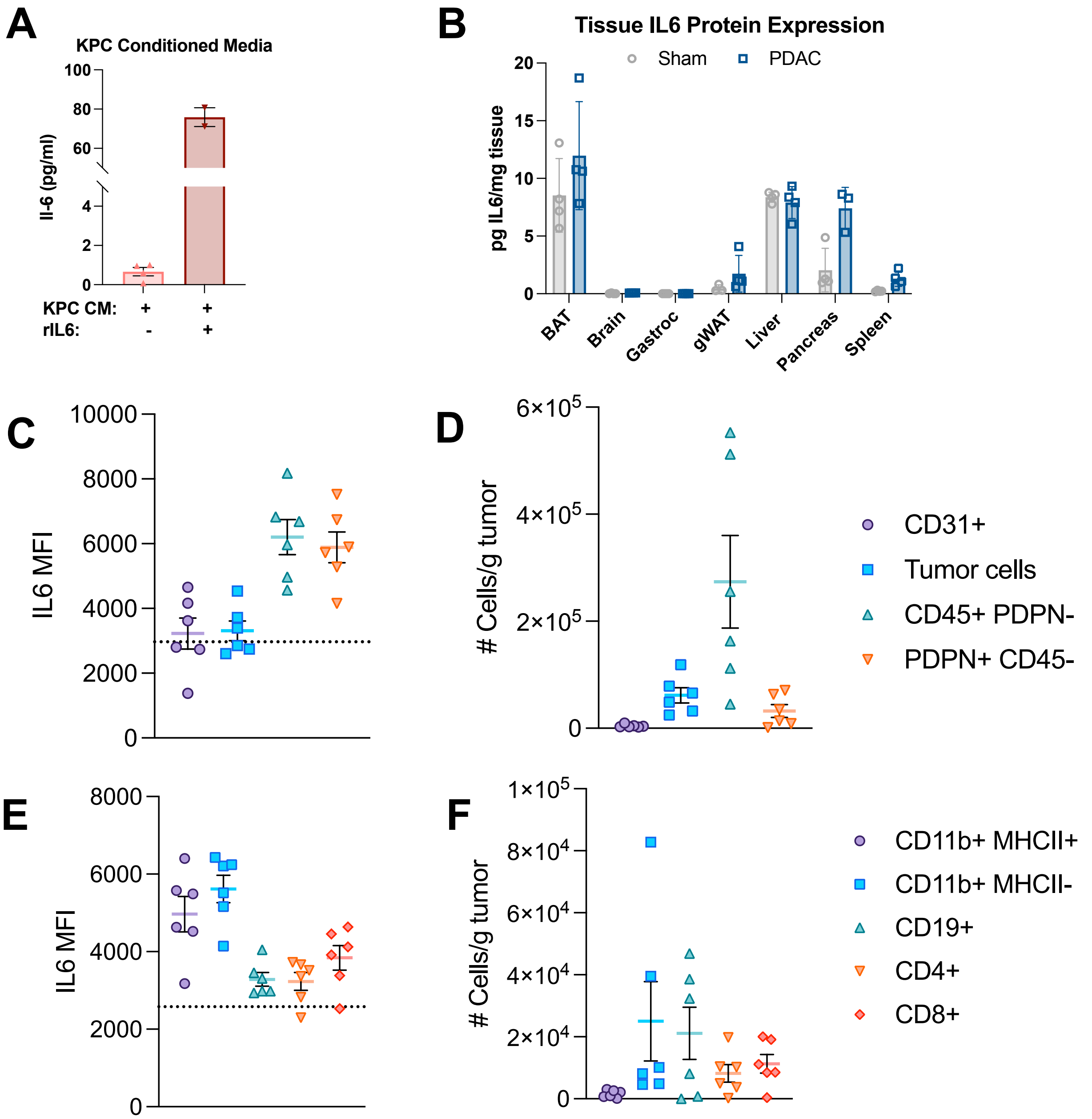

Supplemental Figure 3. IL-6 is derived from the tumor microenvironment (A) IL-6 protein secreted into media of KPC PDAC cells. (B) IL-6 protein concentration in whole protein lysates from BAT, brain, gastrocnemius muscle (no IL-6 detected), gWAT, liver, and spleen. n = 4 male mice per group. (C) Mean fluorescence intensity (MFI) for fluorescently conjugated IL-6 antibody in cell populations isolated from whole pancreas cell suspension. (D) Number of cells per population per gram of tumor, cell number was calculated using CountBright beads. (E) Mean fluorescence intensity (MFI) for fluorescently conjugated IL-6 antibody in CD45+ sub populations isolated from whole pancreas cell suspension. (F) Number of cells per population per gram of tumor, cell number was quantified using CountBright beads. Cell populations are defined as endothelial cells (CD31+), tumor cells (CD90.1+), immune cells (CD45+, PDPN-), and cancer associated fibroblasts (PDPN+ CD45-). Immune cell populations (panels E-F) are within the CD45+ population. (C-F) n = 6 male PDAC mice. Error bars represent SEM. A-D statistically tested with multiple unpaired t tests with Holm-Sidak multiple testing correction. **** p<0.0001, ***p<0.001, **p<0.01, *p<0.05.

Supplemental Figure 4

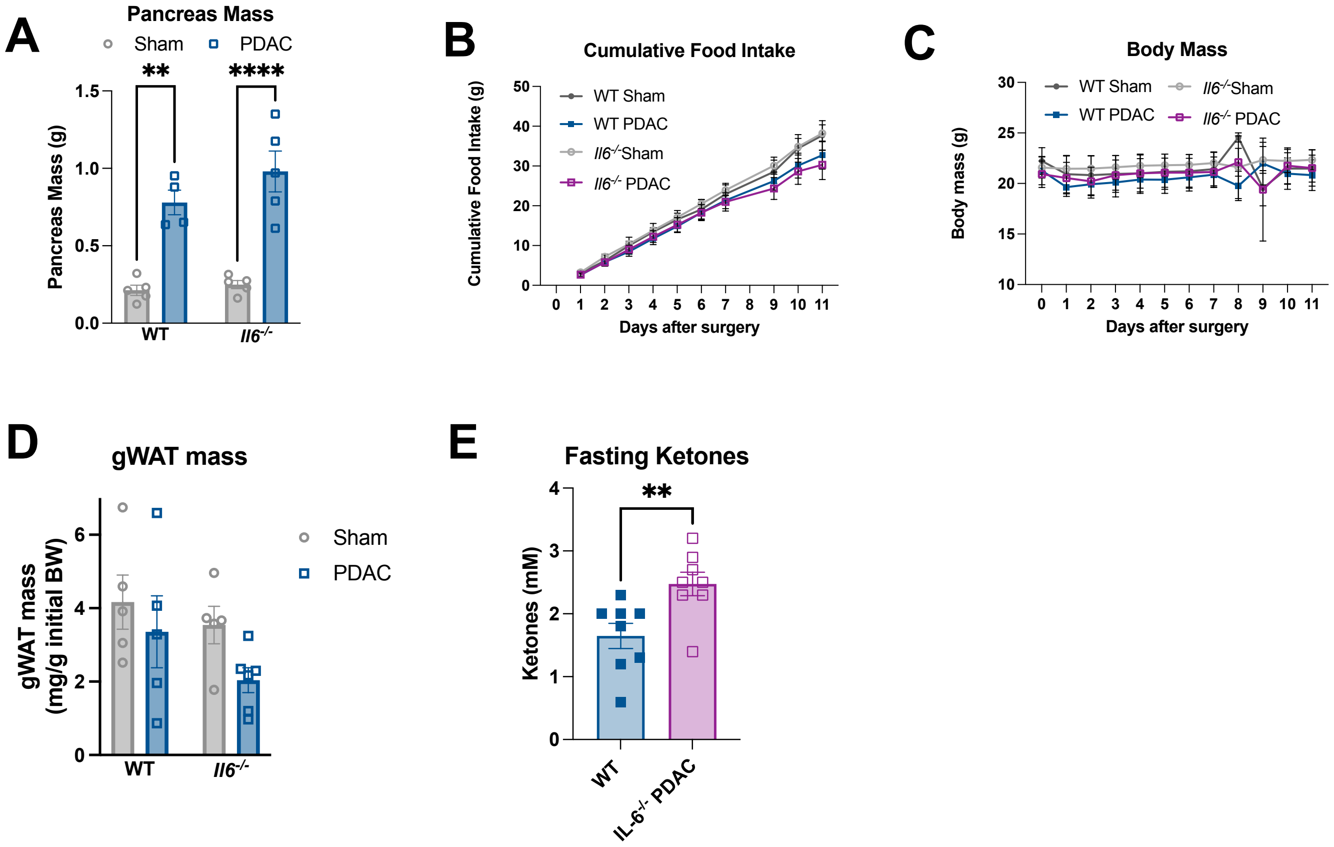

Supplemental Figure 4. Physiology of IL-6$^{-/-}$ mice. (A) Pancreas/tumor mass at euthanasia. n = 2 female, 3 male (sham/WT); 2 female, 3 male (sham/IL-6-/-); 3 female, 2 male (PDAC/WT); 3 female, 3 male (PDAC/IL-6-/-). (B) Cumulative food intake, (C) and body mass over study. (D) gWAT mass at termination, normalized to initial body weight. n = 2 female, 3 male (sham/WT); 2 female, 3 male (sham/IL-6-/-); 3 female, 2 male (PDAC/WT); 3 female, 3 male (PDAC/IL-6-/-). (E) Fasting blood ketone levels at euthanasia. Pairwise comparisons tested with parametric, unpaired t-test. **** p<0.0001, ***p<0.001, **p<0.01, *p<0.05. Independent biological replicates are indicated by individual points on bar graphs, unless noted otherwise.

Supplemental Figure 5

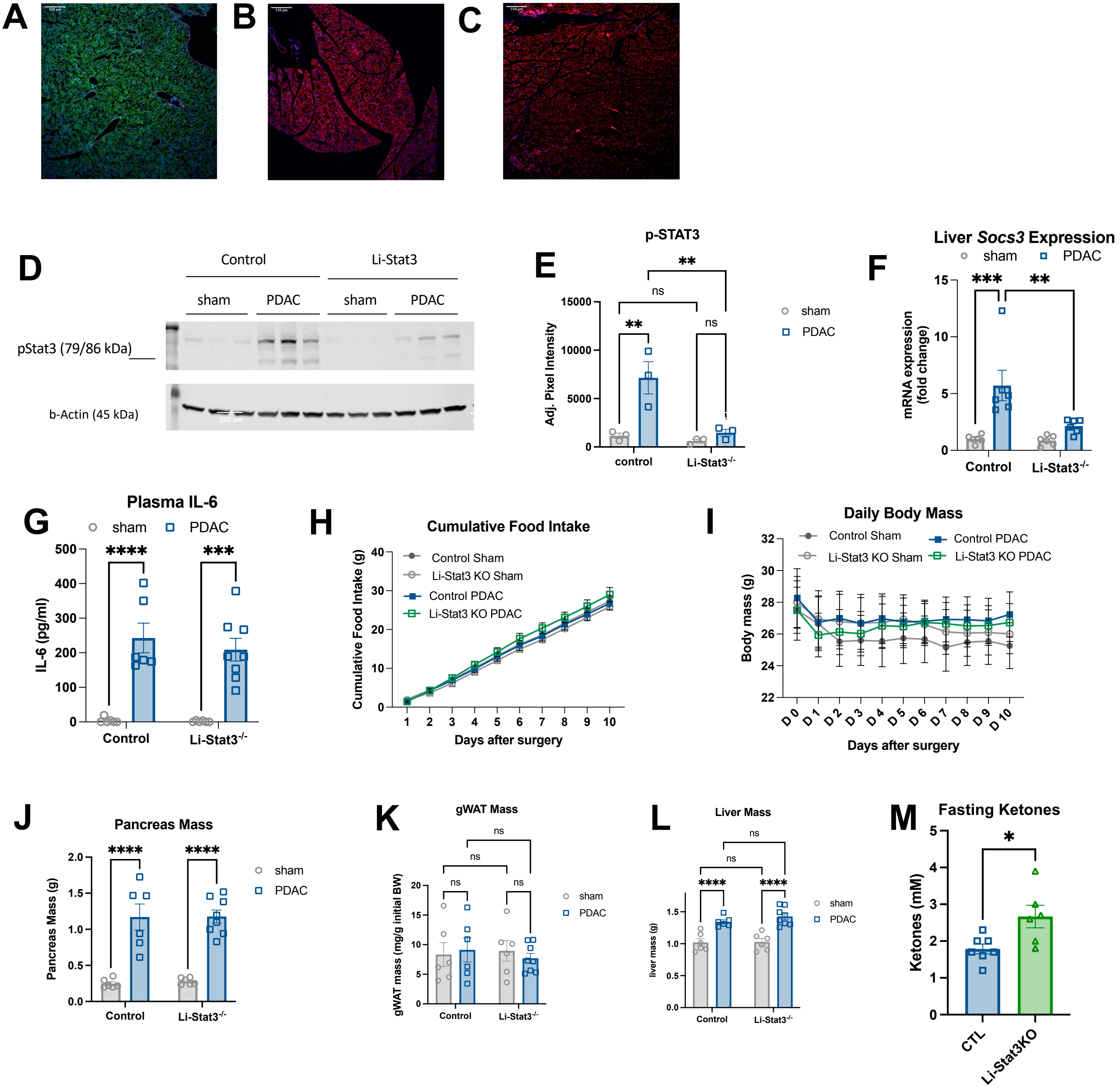

Supplemental Figure 5. Hepatocyte STAT3 KO is tissue specific. (A-C) 3-channel overlay fluorescent images of liver (A), pancreas (B), and gastrocnemius muscle (D) from ROSA$^{mT/}$mG;Albumin-Cre mice, which expresss cell membrane-localized tdTomato in the absence of cre recombinase, and cell membrane-localized EGFP in the presence of cre recombinase. Green is EGFP, red is tdTomato, and blue is DAPI. Scale bar depicts 150 um. (D)Western blot for p-STAT3 (top) and b-actin (bottom). (E) Densitometry analysis of p-STAT3 western blot in A, normalized to b-actin expression. (F) qPCR analysis of Socs3 expression in whole liver of WT and Li-Stat3-/- mice. (G) Plasma IL-6 concentration at euthanasia. (H) Cumulative food intake over 10 days of study. (I) Body mass over course of study. (J) Pancreas mass at termination of PDAC and sham animals. (K) Gonadal white adipose tissue mass at termination, normalized to initial body weight. (L) Liver mass at termination, normalized to initial body weight. (M) Fasting ketones in PDAC mice as baseline for octanoate challenge. (A-B, H-K) n = 3 female, 3 male (sham/control); 2 female, 4 male (sham/ Li-Stat3-/-); 2 female, 4 male (PDAC/control); 3 female, 5 male (PDAC/ Li-Stat3-/-). (C-E) representative images based on n = 3 male mice. Error bars represent SEM. All analyses for 2x2 studies were statistically tested with a full effects model 2-way ANOVA and Sidak multiple comparisons test. **** $p<0.0001$, *** $p<0.001$, ** $p<0.01$, * $p<0.05$.

Supplemental Figure 6

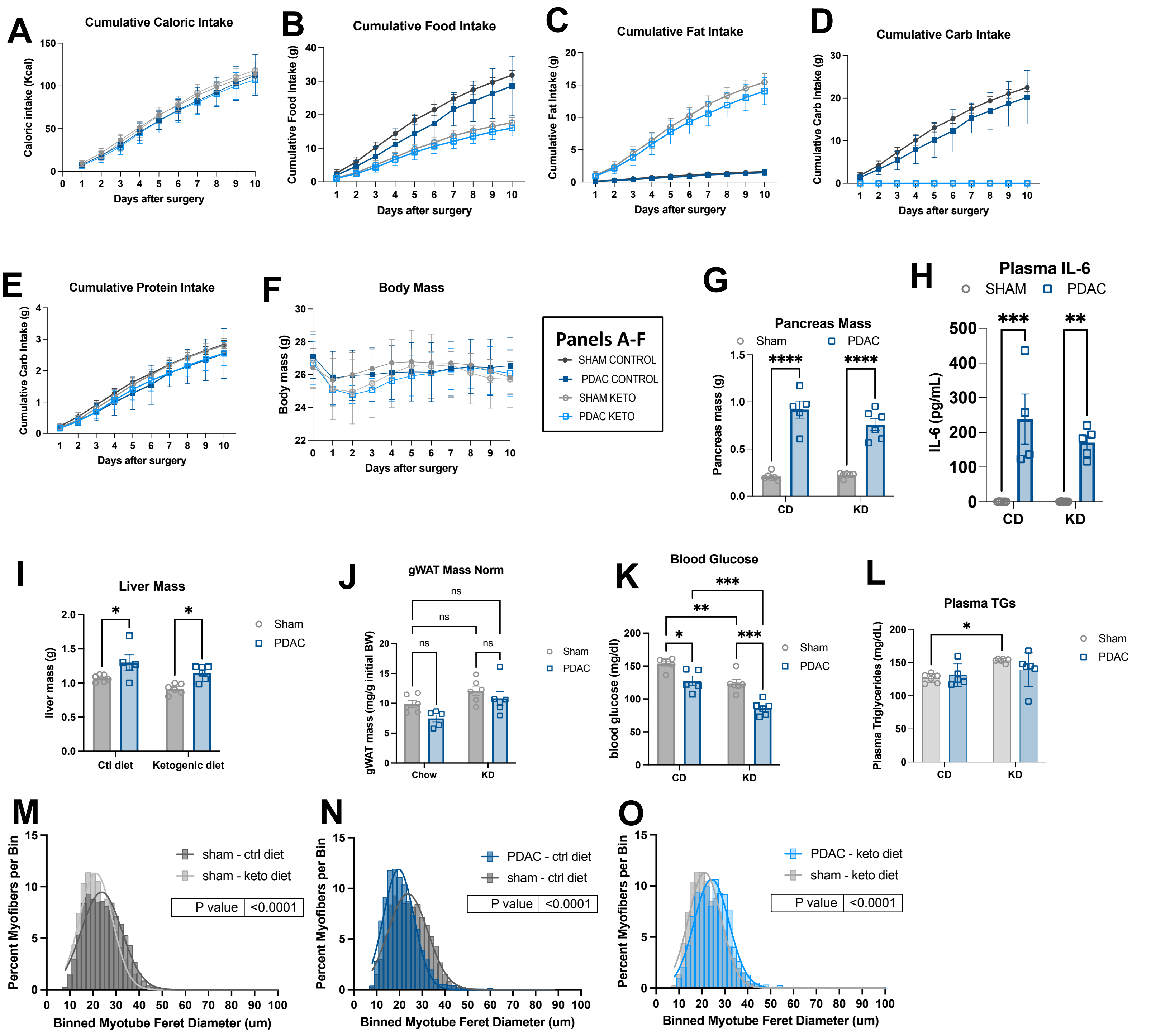

Supplemental Figure 6. Nutrient intake and physiology of mice on ketogenic diet. (A-E) Cumulative nutritional intake for mice fed control diet and ketogenic diet: caloric (Kcal) (A), food mass (g) (B), fat (g) (C), carbohydrate (g) (D), protein (g) (E). (F) Longitudinal body mass (g). (G) Pancreas/tumor mass. (H) Plasma IL-6 levels. (I) Liver mass at termination. (J) Gonadal white adipose tissue (gWAT) mass at termination normalized to initial body weight. (K) Blood glucose levels at termination. (L) Plasma triglycerides at termination. (M-O) Quantification of minimum feret diameter of myofibers from PDAC and sham mice mice fed control or ketogenic diet. Feret diameters were binned to a histogram and fit with a non-linear regression (Gaussian, least squares regression). P values represented on graph were tested by extra sum-of-squares F test. (M-O) n = 6 male mice (sham/CD, sham/KD, PDAC/KD), 5 male mice (PDAC/CD). Error bars represent SEM. All analyses for 2x2 studies were statistically tested with a full effects model 2-way ANOVA and Sidak multiple comparisons test. **** p<0.0001, ***p<0.001, **p<0.01, *p<0.05.

	Component Percentage by Weight	
**Component**	**Ketogenic Diet**	**Control Diet**
Carbohydrate	0%	70.8%
Fat	67.7%	5.1%
Protein	15.9%	8.9%

Supplemental Table 2. Component by weight for ketogenic and control diets.

Component	g/Kg
Casein	180
DL-Methionine	2.88
Vegetable Shortening, hydrogenated (Crisco)	440
Cocoa Butter	150
Corn Oil	85
Cellulose	59.1884
Vitamin Mix, AIN-93-VX w/Cellulose (110068)	27
Thiamin (81%)	0.018
Vitamin K1, phylloquinone	0.0036
Choline Bitartrate	4.5
Mineral Mix, w/o Ca & P (98057)	24.1
Calcium Phosphate, dibasic	17.64
Calcium Carbonate	9.54
TBHQ, antioxidant	0.13

Supplemental Table 3. Complete description of components included in ketogenic diet (g component/Kg total food).

Supplemental Figure 7

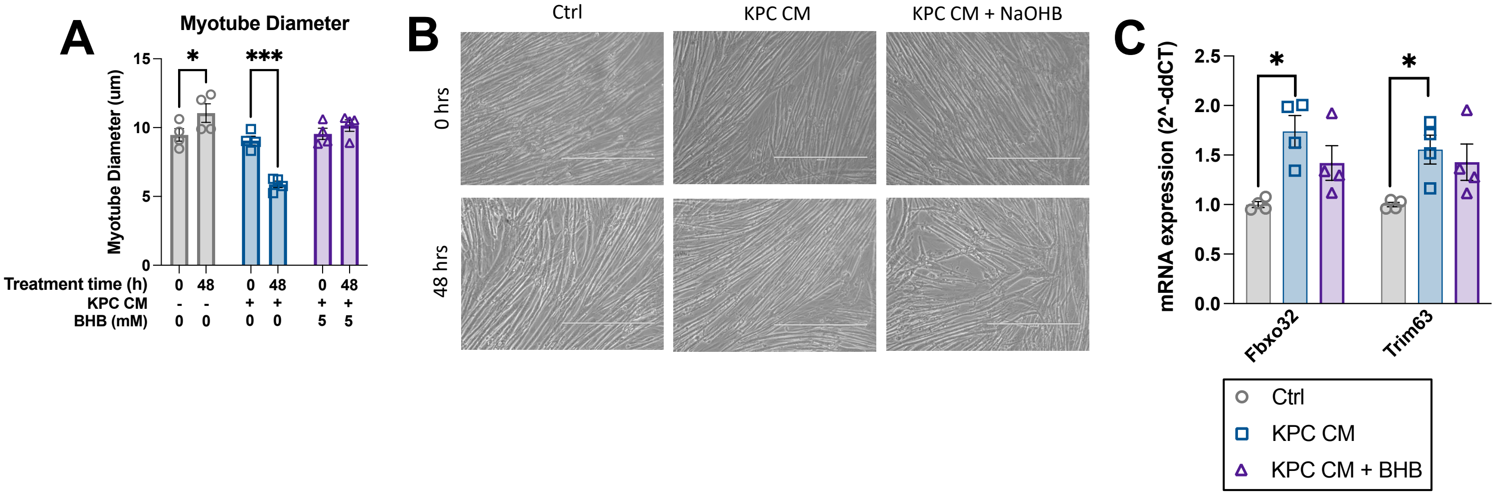

Supplemental Figure 7. Ketones prevent myotube atrophy *in vitro*. (A) C2C12 myotube diameter at baseline and after 48 h exposure to KPC conditioned media (KPC CM) ± sodium beta hydroxybutyrate (BHB). n = 4 wells from 6-well plates per group. (B) Phase contrast images of C2C12 myotubes at 0 and 48 hours in indicated media treatments. Scale bar = 400 um. (C) qPCR analysis of E3 ubiquitin ligases Trim63 and Fbxo32 in C2C12 myotubes 48 hours after media change. Error bars represent SEM. All analyses for 2x2 studies were statistically tested with 2-way ANOVA with Tukey correction for multiple comparisons. 3 and 4-group analyses tested with one-way ANOVA and Tukey correction for multiple comparisons. **** p<0.0001, ***p<0.001, **p<0.01, *p<0.05. Independent biological replicates are indicated by individual points on bar graphs.

Protein	Host	Target	Supplier	Cat. Number
Laminin	Rabbit	Mouse	Sigma	L9393
Myosin Heavy Chain 1E	Mouse	Mouse	DSHB	MF20
STAT3	Rabbit	Mouse	Cell Signaling	49045S
p-STAT3	Rabbit	Mouse	Cell Signaling	9131S
IL6 (PE)	Rat	Mouse	BD Bioscience	562050
CD45.2 (BV786)	Mouse	Mouse	BD Horizon	563686
PDPN (clone 8.1.1) (FITC)	Syrian Hamster	Mouse	BioLegend	127415
CD90.1 (clone OX7) (PE-Cy7)	Mouse	Mouse	BD Pharmingen	561404
CD31 (AF700)	Rat	Mouse	BioLegend	102443
EpCAM (APC)	Rat	Mouse	Invitrogen	17-5791-82
CD90.2 (FITC)	Mouse	Mouse	BD Pharmingen	553004
CD19 (BV480)	Rat	Mouse	BD Bioscience	566167
CD11b (BV650)	Rat	Mouse	BioLegend	101239
MHCII (APC Cy7)	Rat	Mouse	BioLegend	107627
CD4 (PerCP Cy5.5)	Rat	Mouse	BD Pharmingen	561115
CD8 (AF700)	Rat	Mouse	BD Pharmingen	557959
Alexa Fluor 488	Goat	Rabbit	Abcam	ab150077
Alexa Fluor 647	Goat	Mouse	Abcam	ab150115
DyLight™ 800 4X PEG	Goat	Rabbit	Cell Signaling	5151P

Supplemental Table 4. Table of antibodies used.

Gene Name	Supplier	Cat. Number	Used as HK gene in:
18S	Thermo	4333760F	Immortalized cells, muscle, liver
acadm	IDT	Mm.PT.58.9004416	
acox1	IDT	Mm.PT.58.505.3784	
Actb	Thermo	4352663	Adipose
Bdh1	IDT	Mm.PT.58.17371426	
Cpt1	Thermo	Mm01231183_m1	
Ehhadh	IDT	Mm.PT.58.31548328	
Fbxo32	IDT	Mm.PT.58.7025875	
Foxo1	IDT	Mm.PT.58.6477586	
G6pc	Thermo	Mm00839363_m1	
Hmgcs2	Thermo	Mm00550050_m1	
Ppara	Thermo	Mm00440939_m1	
Ppargc1a	Thermo	Mm01208835_m1	
Socs3	Thermo	Mm00545913_s1	
Trim63	IDT	Mm.PT.58.32840172	
Ucp1	IDT	Mm.PT.58.7088262	

Supplemental Table 5. Table of qPCR TaqMan probes used.

Gene Name	F Primer	R Primer
Acaa2	CTGTTTGCCTTTCTTCGTCTT	ACAGATACGCCTTGCAGTC
Acadl	GGTGGAAAACGGAATGAAAGG	GGCAATCGGACATCTTCAAAG
Acat1	CCCTGAAGCCAGGAGAGTTC	GGCCCAAGGGAGTCACATTT
Acsl1	ATCTGGTGGAACGAGGCAAG	TCCTTTGGGGTTGCCTGTAG
Cidea	GTCGCCAAGGTCGGGTCAAGTC	AAAGGGCGAGCTGGATGTATGAGG
Ddit4	CCTGCGCGTTTGCTCATGCC	GGCCGCACGGCTCACTGTAT
Echs1	AGCCTGTAGCTCACTGTTGTC	ATGTACTGAAAGTTAGCACCCG
Hmgcl	GTGAAGATGGCGTCAGTGAG	GGGGTGGGTACAATACTCTTT
Pparg	CGGTTTCAGAAGTGCCTTG	GGTTCAGCTGGTCGATATCAC
Prdm16	GCCATGTGTCAGATCAACGA	CCTTCTTTCACATGCACCAA

Supplemental Table 6. Table of qPCR Sybr primers used.

SUPPLEMENTAL METHODS

Analysis of human CT scans

Scans were manually segmented to include all paraspinal and abdominal wall muscles by a single trained image analyst using Slice-O-Matic software (v.4.3; Tomovision) with verification from a board-certified radiation oncologist. Skeletal muscle cross sectional areas were normalized to the square of height in meters and reported as the skeletal muscle index (SMI). Patients were classified as sarcopenic if SMI < 52.4 cm^2/m^2 for men or < 38.5 cm^2/m^2 for women based on prior reports suggesting prognostic utility of this dichotomization(S15-S16). A convenience sample of sarcopenic and non-sarcopenic patients with PDAC was selected based on the availability of fasting plasma samples collected at the time of surgery. Fasting was defined as patients whose surgeries began prior to 0900 h, as all patients were instructed to take nothing by mouth for at least 8 hours prior to surgery, and compliance was assumed to be highest for surgeries scheduled early in the morning. Control samples were collected from individuals without known malignancy who have either germline genetic mutations conferring elevated risk of developing PDAC or pancreatic cysts. Samples were collected at the time of esophagogastroduodenoscopy as part of pancreatic cancer surveillance. Patients were instructed to take nothing by mouth for 4 h prior to endoscopy, and only samples from procedures scheduled prior to 0900 h were used in analysis.

C2C12 Cell Culturing

We grew C2C12 cells (ATCC CRL-1772) on tissue culture-treated dishes in growth media consisting of high glucose DMEM media (Gibco) supplemented with 10% FBS (Corning) and 1% penicillin/streptomycin (Gibco). Differentiation media (DM) contains 2% horse serum (Corning) in place of 10% FBS. To differentiate C2C12 cells, we changed media to DM, the following day added 50% more DM, and the third day changed media to fresh DM. To complete

atrophy assays, on the fourth day, we changed DM to KPC CM (see above), or, as “C2C12 CM control,” left the DM on the cells. For ketone supplementation studies, CM was supplemented with sodium β-hydroxybutyrate (Sigma) at the indicated concentrations.

Orthotopic PDAC implantation in mice

A vial of frozen KPC cells was thawed prior to each implantation, and 1 million cells were implanted in 23 uL of PBS per animal. All animals were anesthetized with isoflurane, scrubbed with betadine, and a para-midline incision was made in the abdomen to expose pancreas. KPC cells or vehicle (PBS) were injected directly into the pancreatic parenchyma. Pancreas was placed back into position and incision was closed using two sutures (4-0 Polysorb) and two skin staples.

Mouse tissue collection

Blood was collected during cardiac puncture and stored on ice in EDTA-treated tubes for 30 min prior to centrifugation at 1,500 x g for 15 minutes. Plasma was collected, snap frozen in liquid nitrogen, and stored at -80ºC. After euthanasia, we trans-cardially perfused animals with ice-cold PBS. Then, tissues were dissected and weighed prior to flash freezing in liquid nitrogen, or formalin fixation for histological analysis.

Engineered KPC

KPC cells expressing the surface marker Thy1.1 (CD90.1), blasticidin resistance (BSR), and IL-6 were generated from our stock of KPC cells (female) described above. Platinum-E ecotropic packaging cells were transfected with plasmid DNA encoding MSCV-Ova-T2A-Thy1.1 or MSCV-Thy1.1 as described previously (S17). Retroviral supernatants were collected 48 hours following Lipofectamine-mediated transfection, and spun at 2000g for 2 hours at 32°C onto Retronectin (Takara bio) coated 6 well non-tissue culture treated plates. Supernatant was removed and KPC parental tumor cells were plated at 2e6 cells/well. KPC cells were incubated on virus-coated

plates for 24 hours and then removed to standard tissue culture flasks containing DMEM media (Gibco) supplemented with 10% FBS (Corning). Two days later, KPC cells were placed in blasticidin-containing (5ug/mL) complete DMEM media to select for transduced cells. Following antibiotic selection, successful transduction was confirmed via flow cytometry staining for Thy1.1. KPC-BSR cells were implanted for OT-PDAC as described for parental KPC cells. Continued culturing of KPC-BSR cells was done in selection media described.

Brefeldin A injections

For intracellular cytokine staining for flow cytometry, we followed previously published protocols for Golgi transport blockade (3). Briefly, each mouse received 0.25 mg Brefeldin A (Selleckchem) injected via tail vein 6 hours prior to tissue collection.

Cell imaging and quantification

We collected phase-contrast images of C2C12 myotubes at the start and completion of atrophy assays using an AMG EVOS fl cell imaging system. In Fiji (S18), we quantified myotube atrophy by measuring myotube diameter at 3 points across each myotube, for a total of at least 15 myotubes per treatment. Triplicate measures were averaged to achieve one average diameter per myotube, and these values were averaged to provide a single value per treatment. Experimenters were blinded to condition for image analysis.

Cell collection for downstream applications

For transcript (qPCR) downstream analysis, we washed C2C12 cells 2 times with PBS, then scraped cells in an adequate volume of PBS to cover the tissue culture plate. We transferred scraped cells into microfuge tubes and spun at 0.7 G x 7 minutes. Pellets were stored at -80°C.

Conditioned media collection (KPC CM)

We plated KPC cells at 50,000 cells per cm^2, and allowed them to adhere overnight. The following day, we washed with PBS (Gibco) and changed the media to C2C12 differentiation media (DMEM, 2% HS, 1%P/S). 24 hours after the media change, we collected the KPC CM,

centrifuged it at 1100 RPM for 6 minutes, then sterile filtered through a 0.22 um filter. KPC CM was either used immediately or frozen at -80°C.

Fecal Analyses

Fecal protein and lipid content

Sham and PDAC mice were individually housed and allowed *ad libitum* access to food for 7 days after orthotopic injection. Mice were placed in clean cages on day 5. On day 7 feces were collected from cage bottom. To assess total fecal protein, 10 mg of feces was resuspended in 500uL lysis buffer (2% SDS, 150 mM NaCl, 0.5 M EDTA), sonicated, and centrifuged at 10,000 rpm for 15 mins at 4ºC. Protein concentration was assessed using Pierce BCA. To assess total fecal lipid content, 1g feces were pulverized using a pepper grinder, resuspended in 5mL PBS, and lipids were extracted using a modification of the Folch method (S19-S20). Resuspended feces were extracted using choloform:methanol and centrifugation at 1,300 x g for 20 min at RT. Organic phase containing the extracted lipids was collected, and fecal weight was measured after evaporation.

Fecal protease activity

Fecal protease activity was measured as previously described (4). Fresh feces were collected from the distal colon at the time of sacrifice, 7 days after orthotopic injection. Feces were suspended at 10 mg/mL in Protein Buffer A (0.1% Triton X-100, 0.5-M NaCl, 100-mM $CaCl_2$), homogenized and sonicated, then centrifuged at 14,000 RPM for 15 min. 100 uL of supernatant was added to 200 uL of 3% azocasein, and incubated at 37ºC for 1 h. 500 uL of 8% tri-chloroacetic acid was added to each sample, vortexed, centrifuged at 9,000 RPM for 5 min, and the supernatant was assayed for absorbance at 366 nm.

Flow Cytometry

Sample preparation

We collected tumors from mice 10 days post implantation, and tumors were weighed, then

placed in PBS on ice. Tumor tissue was minced and digested (S21). After dissociation, we strained tumor suspension through at 100 um filter, and performed ACK lysis.

Staining

We stained samples with live/dead stain (1:2000) and surface protein antibodies listed in figure legends (1:200 each), and incubated for 20 minutes room temperature. After staining, we washed samples with FACS buffer and pelleted. For intracellular staining (IL-6), we resuspended cells in antibody diluted 1:200 in FACS buffer and incubated at 4°C overnight. The next day we washed and resuspended cells with FACS buffer prior to analysis.

Instrumentation and analysis

All samples were analyzed using the Cytek Aurora flow cytometer (Cytek Biosystems), data was analyzed in FlowJo™ v10.8.1.

Lipid analysis

Blood, liver, and gastrocnemius tissue were collected from PDAC and sham mice fed *ad libitum* or fasted for 24 hours and terminated at 7 and 10 days after tumor implantation. Plasma NEFA and TG concentrations were measured using kits (NEFA: Wako Diagnostics 999–34691, 995–34791, 991–34891, and 993–35191; TG: StanBio #2100). For liver and gastrocnemius, lipids were first extracted using a modification of the Folch method (5). Lipids were extracted in chloroform:methanol (1.4:1), centrifuged at 3,000 RPM for 20 min Organic phase containing the extracted lipids was collected, and crude lipid weight was measured after evaporation in a speedvac. To measure NEFAs and TGs, lipid pellets were resuspended in 10 uL chloroform, then suspended in 100 uL PBS. NEFA and TG concentration were measured using the kits described above, and final concentration was normalized to tissue input.

Murine indirect calorimetry

Oxygen consumption (VO_2), carbon dioxide production (VCO_2), respiratory exchange ratio (RER), and heat production were measured by indirect calorimetry (Oxymax, Columbus

Instruments, Columbus, OH, USA). Mice were housed in separate chambers at 26 ± 1ºC and acclimatized to the chambers for 8 hours a day for 2 days prior to conducting the study. Starting 2 days prior to tumor implantation, mice were placed in the chambers for 27 hours at a time, from 0900 to 1300 h the following day. Recordings were made on days -2, 2, 6, and 9 after tumor implantation. Mice were fed *ad libitum* through the first three days after tumor implantation, then were restricted to 50% of their baseline food intake. Food was placed in cages or chambers at 1700 each day. Samples were recorded every 3 min with room air reference recorded every 30 min and air flow to chambers set at 500 ml/min. Indirect calorimetry measurements reflect periods of movement and inactivity, including both feeding and fasting periods each day.

Oil Red O Staining

Livers from PDAC and sham mice terminated 7 days after orthotopic injection were collected and snap frozen after perfusion with ice cold PBS. 12 um-thick sections were collected via cryostat and stored at -80°C until staining (S22). After equilibration to room temperature, slides were incubated in 1 mL of oil red O working solution at RT for 5 min, then counterstained in Mayer’s hematoxylin for 15 s, before being washed under running water for 30 min, dried, and cover-slipped. Ten bright field images were captured at 20X magnification per sample and quantified in ImageJ using previously described thresholding. Total staining was quantified as integrated density, calculated as the product of *area* and *mean gray value.*

SUPPLEMENTAL REFERENCES

S1. Babic A, Schnure N, Neupane NP, Zaman MM, Rifai N, Welch MW, et al. Plasma inflammatory cytokines and survival of pancreatic cancer patients. Clin Transl Gastroenterol. 2018;9:145.

S2. Fujita J, Tsujinaka T, Yano M, Ebisui C, Saito H, Katsume A, et al. Anti-interleukin-6 receptor antibody prevents muscle atrophy in colon-26 adenocarcinoma-bearing mice with modulation of lysosomal and ATP-ubiquitin-dependent proteolytic pathways. Int J Cancer. 1996;68:637-43.

S3. Long KB, Tooker G, Tooker E, Luque SL, Lee JW, Pan X, et al. IL6 Receptor Blockade Enhances Chemotherapy Efficacy in Pancreatic Ductal AdenocarcinomaIL6R Blockade Enhances Chemotherapy Efficacy. Molecular cancer therapeutics. 2017;16:1898-908.

S4. Puchalska P, Crawford PA. Multi-dimensional Roles of Ketone Bodies in Fuel Metabolism, Signaling, and Therapeutics. Cell Metab. 2017;25:262-84.

S5. Rothman DL, Magnusson I, Katz LD, Shulman RG, Shulman GI. Quantitation of hepatic glycogenolysis and gluconeogenesis in fasting humans with 13C NMR. Science. 1991;254:573-6.

S6. Lowell BB, Goodman MN. Protein sparing in skeletal muscle during prolonged starvation. Dependence on lipid fuel availability. Diabetes. 1987;36:14-9.

S7. Prado CMM, Lieffers JR, McCargar LJ, Reiman T, Sawyer MB, Martin L, et al. Prevalence and clinical implications of sarcopenic obesity in patients with solid tumours of the respiratory and gastrointestinal tracts: a population-based study. The Lancet Oncology. 2008;9:629-35.

S8. Foley K, Rucki AA, Xiao Q, Zhou D, Leubner A, Mo G, et al. Semaphorin 3D autocrine signaling mediates the metastatic role of annexin A2 in pancreatic cancer. Science signaling. 2015;8:ra77-ra.

S9. Falconer JS, Fearon K, Plester CE, Ross JA, Carter DC. Cytokines, the acute-phase response, and resting energy expenditure in cachectic patients with pancreatic cancer. Annals of surgery. 1994;219:325.

S10. Parrilla R. Flux of metabolic fuels during starvation in the rat. Pflugers Archiv: European Journal of Physiology. 1978;374:3-7.

S11. McGarry JD, Foster DW. The regulation of ketogenesis from octanoic acid. The role of the tricarboxylic acid cycle and fatty acid synthesis. J Biol Chem. 1971;246:1149-59.

S12. Thibaut MM, Sboarina M, Roumain M, Pötgens SA, Neyrinck AM, Destrée F, et al. Inflammation-induced cholestasis in cancer cachexia. Journal of cachexia, sarcopenia and muscle. 2021;12:70-90.

S13. Suh S-Y, Choi YS, Yeom CH, Kwak SM, Yoon HM, Kim DG, et al. Interleukin-6 but not tumour necrosis factor-alpha predicts survival in patients with advanced cancer. Supportive Care in Cancer. 2013;21:3071-7.

S14. Oliff A, Defeo-Jones D, Boyer M, Martinez D, Kiefer D, Vuocolo G, et al. Tumors secreting human TNF/cachectin induce cachexia in mice. Cell. 1987;50:555-63.

S15. Grossberg AJ, Chamchod S, Fuller CD, Mohamed AS, Heukelom J, Eichelberger H*, et al.* Association of body composition with survival and locoregional control of radiotherapy-treated head and neck squamous cell carcinoma. JAMA oncology **2016**;2(6):782-9.

S16. Prado CM, Lieffers JR, McCargar LJ, Reiman T, Sawyer MB, Martin L*, et al.* Prevalence and clinical implications of sarcopenic obesity in patients with solid tumours of the respiratory and gastrointestinal tracts: a population-based study. The lancet oncology **2008**;9(7):629-35.

S17. Eil R, Vodnala SK, Clever D, Klebanoff CA, Sukumar M, Pan JH*, et al.* Ionic immune suppression within the tumour microenvironment limits T cell effector function. Nature **2016**;537(7621):539-43.

S18. Schindelin J, Arganda-Carreras I, Frise E, Kaynig V, Longair M, Pietzsch T*, et al.* Fiji: an open-source platform for biological-image analysis. Nature methods **2012**;9(7):676-82.

S19. Folch J, Lees M, Sloane Stanley GH. A simple method for the isolation and purification of total lipids from animal tissues. J biol Chem **1957**;226(1):497-509.

S20. Kraus D, Yang Q, Kahn BB. Lipid extraction from mouse feces. Bio-protocol **2015**;5(1):e1375-e.

S21. Helms EJ, Berry MW, Chaw RC, DuFort CC, Sun D, Onate MK*, et al.* Mesenchymal lineage heterogeneity underlies nonredundant functions of pancreatic cancer–associated fibroblasts. Cancer discovery **2022**;12(2):484-501.

S22. Mehlem A, Hagberg CE, Muhl L, Eriksson U, Falkevall A. Imaging of neutral lipids by oil red O for analyzing the metabolic status in health and disease. Nature protocols **2013**;8(6):1149-54.
